# Clinical and biochemical characteristics of patients with ornithine transcarbamylase deficiency and in silico analysis of *OTC* gene

**DOI:** 10.1186/s13023-025-03624-4

**Published:** 2025-03-18

**Authors:** YinChun Zhang, Xia Gu, Congcong Shi, Hui Xiong, DongFan Xiao, ZhiRong Deng, Lu Wang, XiMei Yang, Tao Wei, PuPing Liang, Hu Hao

**Affiliations:** 1 Department of Pediatrics, The Sixth Affiliated Hospital, Sun Yat-sen University, No. 26 Yuancun Erheng Road, Tianhe District, Guangzhou, 510655 China; 2 Guangdong Institute of Gastroenterology, The Sixth Affiliated Hospital, Sun Yat-sen University, Guangzhou, China; 3 Inborn Errors of Metabolism Laboratory, The Sixth Affiliated Hospital, Sun Yat sen University, Guangzhou, China; 4 Biomedical Innovation Center, The Sixth Affiliated HospitalSun Yat-sen University, Guangzhou, China; 5 Department of Bioengineering, College of Food Science, South China Agricultural University, Guangzhou, Guangdong 510642 China; 6 MOE Key Laboratory of Gene Function and Regulation, State Key Laboratory of Biocontrol, School of Life SciencesSun Yat-sen University, Guangzhou, Guangdong 510275 China

**Keywords:** Ornithine transcarbamylase defciency, *OTC*, Gene variants, In Silico snalysis

## Abstract

**Background:**

This study seeks to elucidate the clinical and biochemical features of Ornithine transcarbamylase deficiency (OTCD), a pleomorphic congenital hyperammonemia disorder with a non-specific clinical phenotype. Additionally, the research aims to analyze the mutation spectrum of the *OTC* gene and its potential association with phenotype, as well as to perform an in silico analysis of novel OTC variants to elucidate their structure-function relationship.

**Methods:**

In this study, we conducted a retrospective analysis of the clinical and biochemical features of 12 patients with OTCD and examined their metabolite profiles. Additionally, we reviewed existing literature to explore the range of mutations in the *OTC* gene and their possible associations with phenotypic outcomes. Furthermore, we employed the high ambiguity-driven protein-protein docking (HADDOCK) algorithm and protein-ligand interaction profiler (PLIP) to predict the pathogenicity of these mutations and elucidate the underlying mechanisms of pathogenesis in novel variants of the *OTC* gene.

**Results:**

Nine cases, all of which were male, presented with early onset, while two cases, all of which were female, exhibited late onset. Additionally, one male case was asymptomatic. The ages of the patients at the time of diagnosis ranged from 1 day to 12 years. Peak plasma ammonia levels were found to be higher in patients with early onset compared to those with late onset. Molecular analyses identified a total of 12 different mutations, including two novel mutations (V323G and R320P). In silico analysis indicated a potential difference in affinity between wild-type and mutant OTCase, with V323G and R320P mutations leading to a decreased binding ability of OTCase to the substrate, potentially disrupting its function.

**Conclusion:**

This study broadened the genetic variation spectrum of OTCD and provided substantial evidence for genetic counselling to affected families. Additionally, we elucidated variant data of *OTC* in Chinese patients through comprehensive literature review. Given the ongoing uncertainty surrounding the genotype-phenotype correlation of OTCD, the results of our in silico analysis can contribute to a deeper understanding of this complex, rare, and severe genetic disorder.

**Supplementary Information:**

The online version contains supplementary material available at 10.1186/s13023-025-03624-4.

## Introduction

Urea cycle disorders (UCDs) are genetic disorders resulting from mutations in one of five core enzymes, an activating enzyme, or one of two mitochondrial antiporters, leading to errors in ammonia detoxification synthesis. The estimated incidence of UCDs ranges from 1:35,000 to 1:69,000 [1, 2]. However, the actual incidence may be higher due to underdiagnosis, particularly in fatal cases. Ornithine transcarbamylase deficiency (OTCD) is among the most prevalent UCDs, representing approximately half of all inherited urea cycle disorders. The estimated prevalence of OTCD ranges from 1/14,000 to 1/77,000 [3], with no reported incidence in China. OTCD is an X-linked recessive genetic disorder caused by mutations in the human *OTC* gene located on Xp21.1, which spans 73 kb and consists of 10 exons and 9 introns [4]. Biochemically, OTCD disrupts ureagenesis, leading to hyperammonemia. Ornithine transcarbamylase catalyzes the conversion of ornithine and carbamyl phosphate into citrulline, resulting in excess carbamoyl phosphate production that reacts with aspartate to produce excess orotic acid [5]. Consequently, biochemical manifestations in OTCD patients encompass heightened levels of blood glutamine and urinary orotic acid, alongside diminished citrulline levels.

The diversity of variants in the *OTC* gene is extensive, with recurrent sequence variations exhibiting variability based on ethnic backgrounds. Over 500 variants within the 10 exons of the *OTC* gene have been documented. OTCD is now recognized as a highly diverse inherited disorder [6], with a wide range of clinical manifestations. The severity of symptoms experienced by patients is associated with deleterious mutations, although not solely determined by them. Various environmental factors, including infection, high-protein diets, and steroid therapy, have been shown to influence the onset and presentation of the disease [3, 7, 8].

Clinically, nearly all hemizygous males will develop OTCD, while approximately 20% of heterozygous females may exhibit neurocognitive disorders as a result of skewed X-inactivation. The clinical presentation of carrier females with OTCD is characterized by significant variability due to the degree of X-inactivation in hepatocytes [9]. OTCD clinical phenotypes can generally be categorized into two groups: early-onset (onset age ≤ 30 days) and late-onset (onset age > 30 days) or asymptomatic. Common symptoms include vomiting, anorexia, lethargy, seizures, muscle weakness, and coma, which may be triggered or exacerbated by high protein intake, fasting, infections, trauma, surgery, or childbirth [10]. Currently, there is a growing number of reports on patients with OTCD in China; however, there is a lack of comprehensive summary regarding the spectrum of *OTC* gene mutations in the country. The relationships between genotypes and phenotypes are intricate and varied, presenting a challenge in exploring the pathogenic mechanisms of environmental factors on OTCD patients at a genetic or epigenetic level.

While there have been recent advancements in the testing of various OTC gene variants, their clinical implications remain uncertain. It is imperative for practitioners to study the functional implications of *OTC* variants in order to conduct precise risk assessments and offer counseling to individuals with OTCD. Utilizing bioinformatics tools to assess the impact of *OTC* variants on protein stability, interactions with other molecules, and enzymatic activity can offer valuable insights into the functional consequences of these mutations on the *OTC* gene.

## Materials and methods

### Patients and methods

A retrospective analysis was conducted on the clinical presentations, biochemical profiles, and molecular genetic attributes of twelve patients (ten males and two females) diagnosed with OTCD between 2014 and 2024 at the Sixth Affiliated Hospital, Sun Yat-sen University. This research was authorized by the institutional ethics committee and adhered to approved study protocols (2024ZSLYEC-393, 2023-063). Written consent was obtained from the parents of all participants. We selected 12 OTCD patients based on these criteria: Inclusion: ① Clinical diagnosis, ② Positive OTC gene test with pathogenic or likely pathogenic variants, or VUS variants with clinical relevance or functional evidence, ③ Complete clinical data. Exclusion: ① Other severe genetic metabolic disorders, ② Clinical diagnosis lacking OTC gene test, ③ Incomplete core data, ④ Uninterpretable test results.

### Mutant analysis

The high-throughput sequencing data analysis process was used to analyze the sequencing results. Mutation sites were determined by searching the human gene mutation database (HGMD, https://www.hgmd.cf.ac.uk/ac/index.php), online human Mendelian heredity (OMIM, http://omim.org) and Clinvar (http://www.ncbi.nlm.nih.gov/clinvar) databases. Data interpretation rules refer to the relevant guidelines of the American College of Medical Genetics and Genomics (ACMG).

### Review of the literature

Currently, there is a growing number of reports on patients with OTCD in China; however, there is a lack of comprehensive summary on the spectrum of *OTC* gene mutations in the country. To address this gap, we conducted a survey of genetic information pertaining to Chinese OTCD patients by searching databases such as PubMed (https://pubmed.ncbi.nlm.nih.gov), Google Scholar(https://scholar.google.com), China National Knowledge Infrastructure (https://www.cnki.net/), Wanfangdata (https://www.wanfangdata.com.cn/), Chinese medical case repository (https://cmcr.yiigle.com/index), and YIIGLE(https://www.yiigle.com/index) using keywords including “*OTC*”, “OTCD”, “Ornithine transcarbamylase deficiency” and “UCDs” et al. In addition, a comprehensive review of the literature was conducted to examine variations in the *OTC* gene, with specific quotations from 86 relevant papers. Subsequent statistical analysis was performed on various biomarkers including blood ammonia, citrulline, glutamine, urine uric acid, uracil, blood gas analysis, ALT, and AST, stratified by early and late onset groups. The study’s inclusion criteria consisted of Chinese patients with OTCD, complete variant sites in the OTC gene, and relatively comprehensive clinical data. Conversely, exclusion criteria comprised non-Chinese OTCD patients and individuals lacking genetic testing.

### Statistical analysis

Statistical analyses were performed using SPSS 26.0 version (IBM Corporation, Armonk, NY, USA). Analyses using the -Shapiro-Wilk test, histogram, and Q-Q diagram showed that the data were non-normally distributed. Therefore, the values were expressed as medians (quartiles), and an independent sample nonparametric test was performed. *p* < 0.05 was considered statistically significant.

### In silico structural analysis

The protein sequence of *OTC* was retrieved from UniProt (https://www.uniprot.org/uniprotkb/P00480). The X-ray crystallography data for protein structure 1oth.pdb [11] from the Protein Data Bank (PDB) database [12] served as a template structure, and the SWISS-MODEL [13] homology modeling method was utilized to generate both wild-type and mutant *OTC* protein models for enhanced structural accuracy. Furthermore, MutPred2 [14] and PolyPhen-2 [15] were employed to forecast the pathogenicity of the two loci prior to and following mutation. Given the proximity of the two mutation sites to the ligand binding site, the ligand molecule N-(PHOSPHONOACETYL)-L-ORNITHINE (PAO) was docked to both the wild-type and mutant protein structures using AutoDock4 [16]. Subsequently, the protein-ligand interactions pre and post mutation were analyzed utilizing the Protein-Ligand Interaction Profile (PLIP) online server [17]. Subsequently, protein structure simulations were conducted both pre- and post-mutation utilizing the molecular dynamics simulation software GROMACS [18], in conjunction with the force field amber14sb_OL15.ff [19] to examine the impact of mutation on protein stability. Additionally, the online tool Proteins Plus (https://proteins.plus) was utilized to analyze alterations in the volume size of *OTC* protein ligand binding sites following molecular dynamics simulations [20], with visualization and color labeling of protein structures achieved using PyMol [21] and Chimera [22].

## Result

### The clinical characteristics of twelve OTCD patients

Twelve patients diagnosed with ornithine transcarbamylase deficiency (OTCD) were included in the study, with a gender distribution of 10 males and 2 females. The age at diagnosis ranged from 1 day to 12 years. Detailed clinical characteristics of the patients can be found in Table 1, while metabolic findings are presented in Table 2. Among the late-onset patients, all were female, and unfortunately, one patient succumbed to ineffective rescue efforts.

**Table 1:** The clinical characteristics and variants of 12 OTCD patients

Patient NO.	Gender	Age at diagnosis	Type	Clinlcal symptom	OTC Variant Amino acid changes	Clinlcal outcomes
01	M	1D	EO	Hyperammonemia, Hypotonia, Convulsion, Dyspnea	c.512 A > G p.Q171R	Death
02	M	1D	EO	Hyperammonemia, Coma, Hypotonia	c.867 + 1 G > C	Death
03	M	7D	EO	Vomit, Hypotonia, Convulsion, Dyspnea, Coma	c. 782T > C p. I261T	Death
04	F	2Y	LO	Abnormal liver function, Vomit	c.103insA p.V35SerfsX7	Death
05	M	2D	EO	Convulsion, Coma, Dyspnea	c.274 C > T p.R92R*	Death
06	M	2Y6M	AS	Asymptomatic	c.119G > A p.R40H	Alive
07	F	12Y	LO	Dystropy, Breathing attack	c.968T > G p.V323G^△^	Alive
08	M	8D	EO	Hyperammonemia, Metabolic disorder	c. 959G > C p. R320P^△^	Missing
09	M	1 M	EO	Hyperammonemia, Metabolic disorder	c.717 + 1G > T	Alive
10	M	5D	EO	Hyperammonemia, Cyanosis	c.674 C > T p.P225L	Death
11	M	1D	EO	Family history, Hyperammonemia	c.143T > C p.F48S	Death
12	M	3D	EO	Hyperammonemia, Vomit, Coma	c.421 C > T p.R141*	Death

M, Male; F, Female; D, days; M, months; Y, years; EO, early-onset; LO, late-onset; AS, Asymptomatic.“^△^” donates novel mutation

**Table 2:** Metabolic findings of the OTCD patients

Patient NO.	Ammonia (µmol/L)	Citrulline (µmol/L)	Ornithine (µmol/L)	Glutamine (µmol/L)	Alanine (µmol/L)	Arginine (µmol/L)	Orotic acid (mmol/ molCr)	Uracil (mmol /molCr)
01	1250	2.91	96.55	363.42	1432.60	12.76	46.68	1.04
02	750	3.66	21.94	39.46	826.42	12.22	17.80	4.50
03	>700	1.77	45.05	216.48	709.50	11.63	33.11	2.15
04	580	26.20	NA	939.00	NA	17.40	NA	NA
05	>715	2.15	42.63	84.35	845.05	3.46	Elevated	Elevated
06	39	14.9	37.80	396.30	285.10	19.50	NA	NA
07	118	7.16	15.59	40.87	148.74	12.50	1.50	34.20
08	NA	3.72	24.86	36.98	1373.79	11.37	264.32	23.08
09	NA	1.30	56.60	612.90	1992.57	6.03	NA	NA
10	>700	1.64	51.85	112.28	620.88	4.67	NA	NA
11	NA	3.14	121.47	NA	1836.27	10.21	152.40	NA
12	1485	NA	NA	NA	NA	NA	NA	NA

NA, not available; Normal reference values: Ammonia 10 ~ 47 µmol/L; Citrulline 7 ~ 35 umol/L; Ornithine 20 ~ 160µmol/L; Glutamine 6 ~ 30 umol/L; Alanine 70 ~ 350µmol/L; Arginine 3 ~ 50µmol/L; Orotic acid 0 ~ 1.5 mmol/molCr; Uracil: 0 ~ 7 mmol/molCr

### Review of the literature

We conducted a review of 86 papers to analyze the variants of the *OTC* gene in a cohort of 291 Chinese patients with OTCD (Additional file 1). We elucidated 159 variants in *OTC* genes. Our findings indicate that the most prevalent mutations in the Chinese population were c.829 C > T(R277W) (6.53%), c.119G > A(R40H) (6.19%), and c.386G > A(R129H) (3.09%) (Additional file 2, S2-1). Interestingly, the phenotypic manifestations of these mutations, such as R277W and R40H, exhibited significant variability. Specifically, among the patients with these mutations, there were 2 asymptomatic cases (both male), 201 cases of late-onset symptoms (101 female, 99 male, and 1 unspecified gender), and 87 cases of early-onset symptoms (8 female, 76 male, and 3 unspecified gender) (Additional file 2, S2-2).

**Fig. 1:**
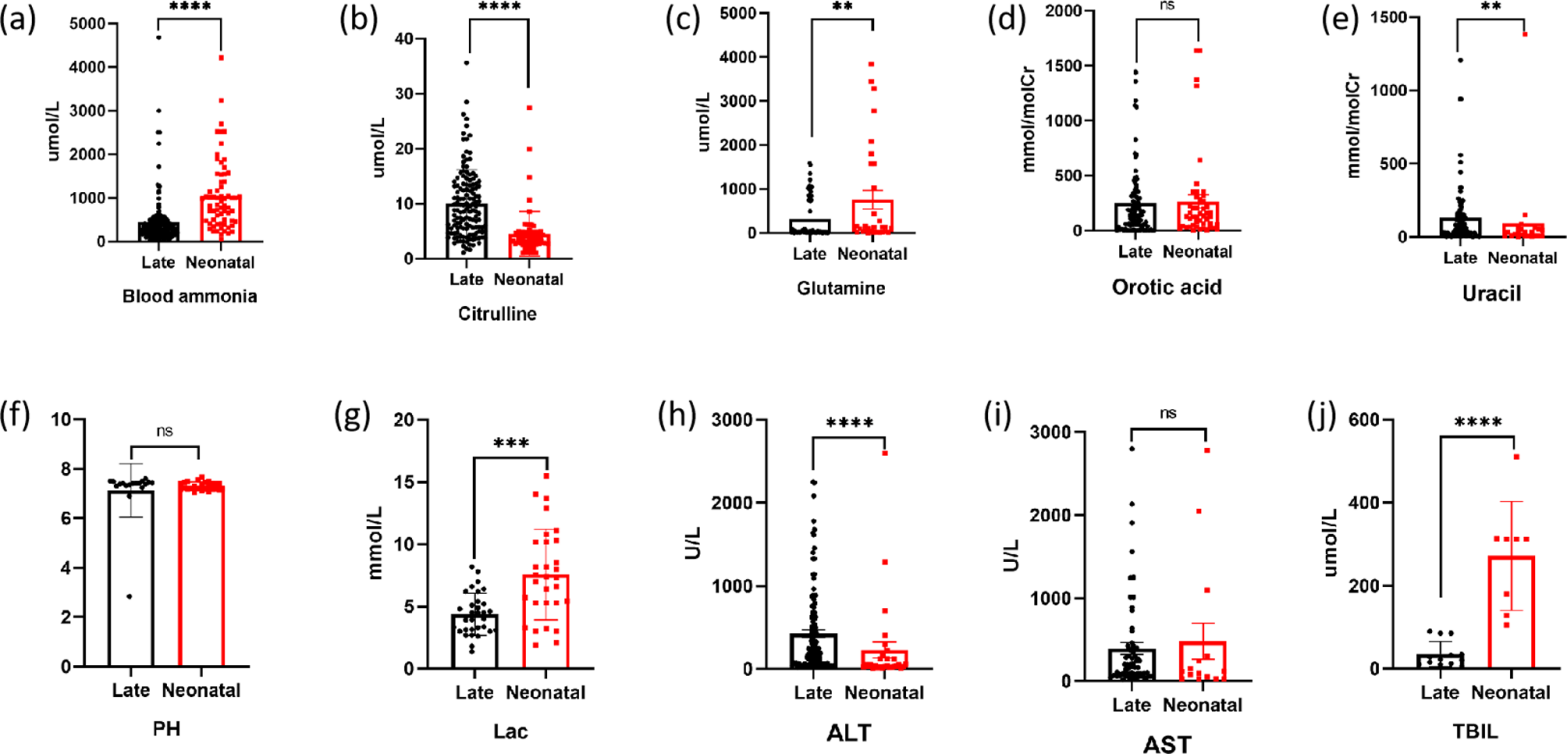
Comparison of biochemical data between diferent phenotypes of OTCD Chinese patients in references. EO, early-onset; LO, late-onset; Lac, lactic acid; ALT, alanine aminotransferase; AST, glutamic-oxalacetic transaminase, TBIL, total bilirubin. **** *P*<0.0001, ***P<0.001, **P<0.01, *P<0.05, ns *P* ≥ 0.05

**Fig. 2:**
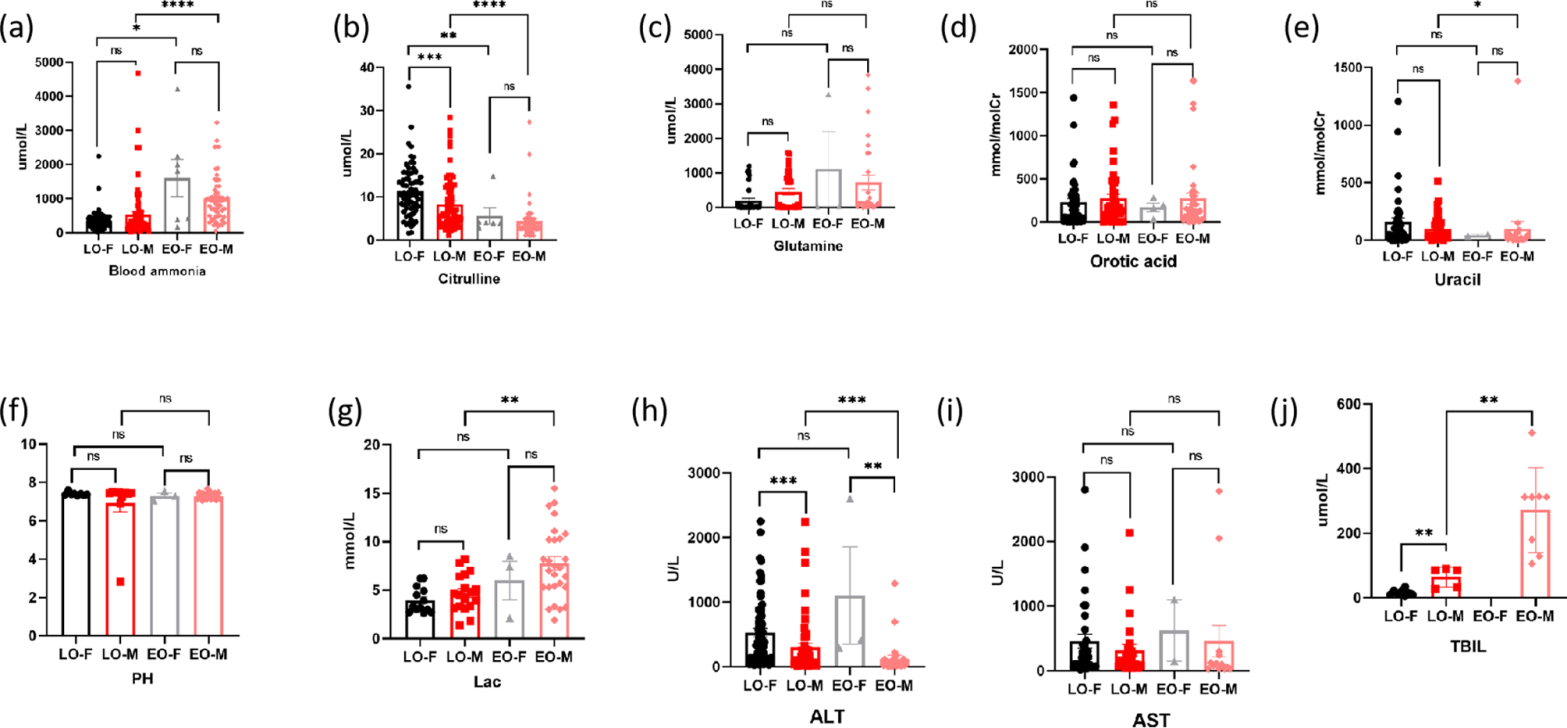
Comparison of biochemical data between diferent phenotypes and gender of OTCD Chinese patients in references. EO, early-onset; LO, late-onset; Lac, lactic acid; ALT, alanine aminotransferase; AST, glutamic-oxalacetic transaminase, TBIL, total bilirubin. **** *P*<0.0001, ***P<0.001, **P<0.01, *P<0.05, ns *P* ≥ 0.05

Based on a statistical analysis of blood ammonia levels, metabolic levels, and biochemical markers in individuals with early-onset and late-onset conditions, it was observed that blood ammonia, glutamine(Fig. 1(a)), lactic acid(Fig. 1(c)), and total bilirubin levels (Fig. 1(g)) were significantly higher in the early-onset group compared to the late-onset group. Conversely, citrulline levels(Fig. 1(b)) were significantly lower in the early-onset group. Additionally, individuals in the early-onset group exhibited milder liver impairment function compared to those in the late-onset group(Fig. 1(h)). We further analyzed whether gender influenced early and late onset clinical phenotypes, we found significant differences in citrulline, ALT and TBIL, and in ALT in late onset patients(Fig. 2, Additional file 2, S2-3).

### OTC protein model construction and conformation analysis

**Fig. 3:**
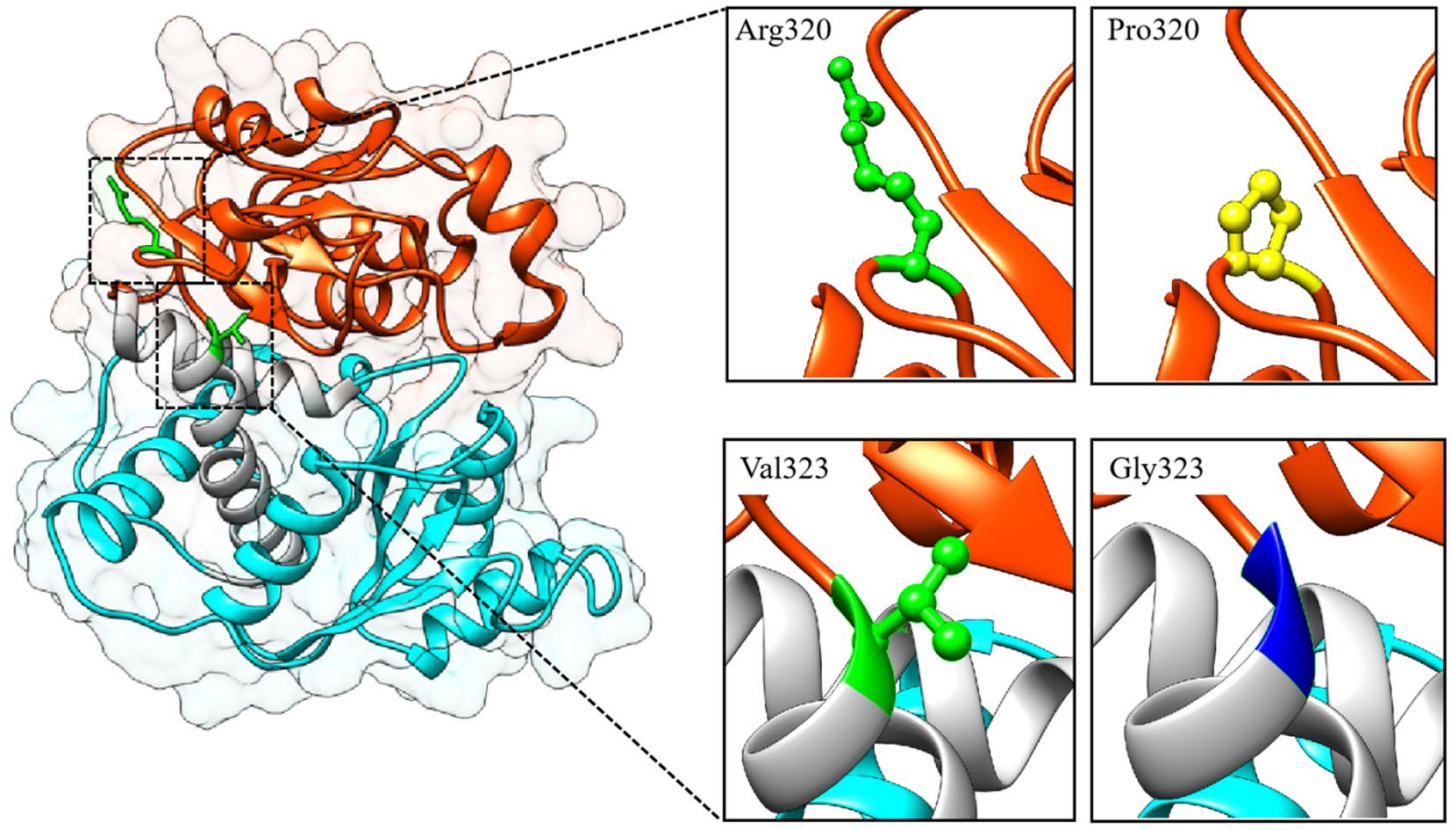
Representations of the wild-type structure of OTC protein and the location of the mutation site. The cyan color labels the ORN-binding/equatorial structural domains and the orange color labels the CP-binding/polar structural domains. In the further enlarged image, the upper part indicates the mutation at site 320 and the lower part indicates the mutation at site 323, where wild-type residues are labeled in green and mutant residues are labeled in yellow and blue

The *OTC* protein consists of a sequence of 354 amino acids. The X-ray resolved structure of the *OTC* gene protein, designated as 1oth.pdb in the Protein Data Bank (PDB) library, binds a PAO molecule. Utilizing this structure as a template, we employed the SWISS-MODEL homology modeling method to generate both wild-type and mutant structures of the *OTC* protein [12, 23, 24]. Previous studies have indicated that OTC proteins exist as trimers, with each subunit containing two distinct domains: a CP binding or polar domain (residues 34–168 and 345–354) and an ORN binding or equatorial domain (residues 183–322) as illustrated in Fig. 3. The protein structure consists of a CP binding or polar domain (residues 34–168 and 345–354) and an ORN binding or equatorial domain (residues 183–322), with an overall topology of each subunit being α/β, characterized by 14 α-helices and 9 β-sheets. The mutation c.959G > C(p.Arg320Pro) is located in helices 11 within the CP binding or polar domain, while the mutation c.968T > G (p.Val323Gly) is not within these two structural domains, but in helices 11. Significantly, the c.968T > G (p.Val323Gly) mutation is situated within the loop region, a secondary structural element that potentially plays a crucial role in preserving the structural integrity of the CP-binding/polar structural domain and the ORN-binding/equatorial structural domain.

### Pathogenicity prediction

MutPred2 and PolyPhen-2 were utilized for the prediction of pathogenicity at two protein loci pre- and post-mutation (Table 3). The MutPred2 software yielded pathogenicity scores of 0.780 for R320P and 0.922 for V323G, surpassing the threshold value of 0.5 and thus classifying them as pathogenic mutations. Additionally, PolyPhen-2 predicted a pathogenicity score of 1 for both R320P and V323G, suggesting that mutations at these loci would result in structural damage to the protein.

**Table 3:** Prediction of pathogenic mutations in R320P and V323G

No.	Nucleotide alteration	Amino acid alteration	Clinical significance	Pathogenicity prediction	
				MutPred2	PolyPhen-2
1	c. 959G > C	p. Arg320Pro	VUS	0.780	1.000
2	c.968T > G	p.Val323Gly	VUS	0.922	1.000

### Protein-ligand interaction prediction

**Fig. 4:**
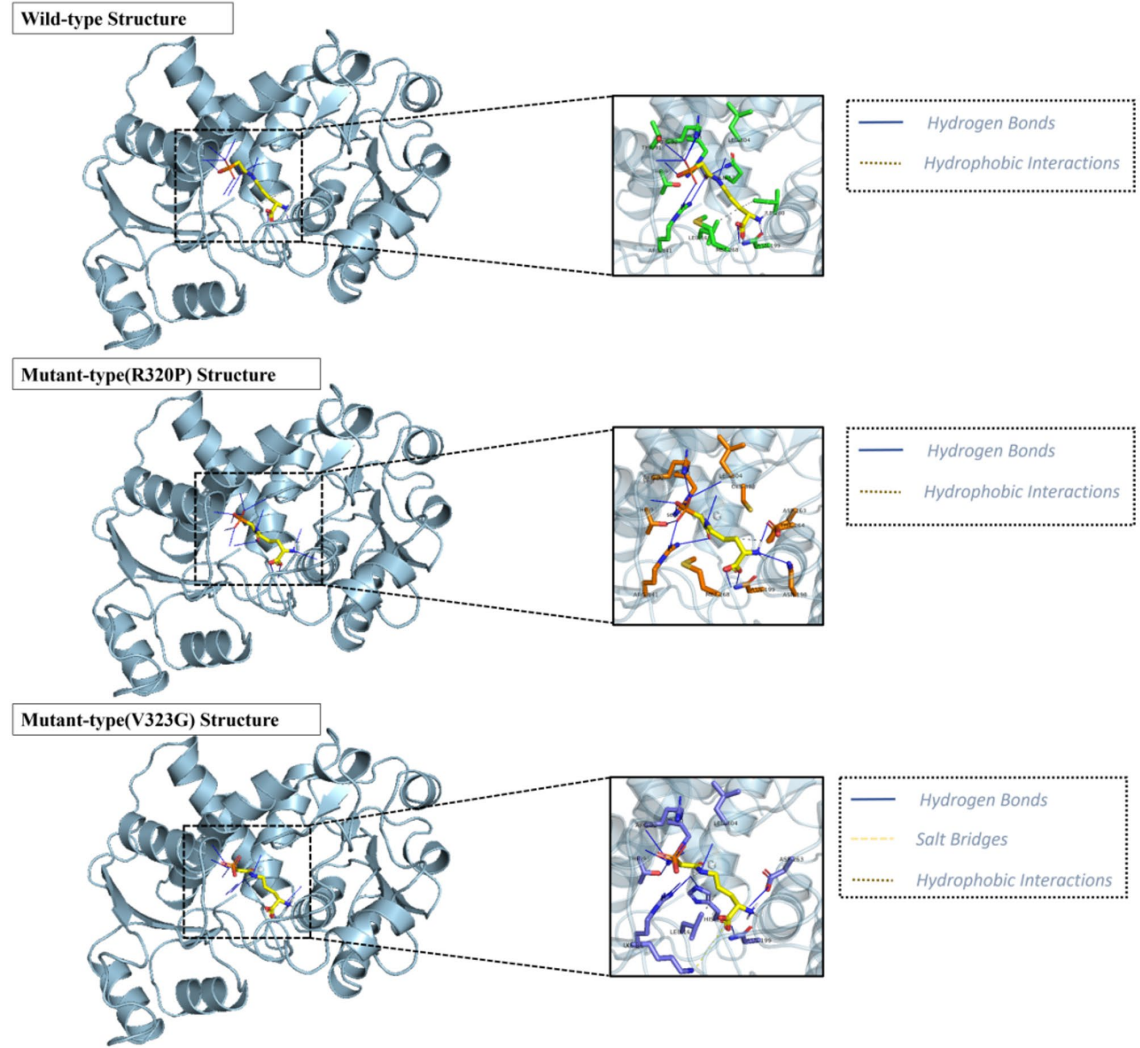
Representations of the protein-ligand interactions of OTC wild-type and mutant-type structures. The PAO molecule was labeled in yellow, interacting residues of wild-type proteins were labeled in green, interacting residues of R320P mutant proteins were labeled in orange, and interacting residues of V323G mutant proteins were labeled in blue

Given the proximity of the two mutation sites to the protein-ligand binding site, molecular docking techniques were employed to investigate the potential impact of these mutations on protein-ligand interactions. Specifically, the ligand PAO bound in the 10th.pdb structure file was docked to both wild-type and mutant protein structures, with the lowest binding free energy (top1) being selected for further analysis (Additional file 2, S2-4). Subsequently, the protein-ligand interactions of the complexes were analyzed using the PLIP online server (Fig. 4). The PLIP analysis revealed that the wild-type protein structure exhibited 11 residues forming 13 hydrogen-bonded interactions and 2 hydrophobic interactions with the PAO. Conversely, the R320P mutant protein displayed 11 residues forming 13 hydrogen-bonded interactions and 1 hydrophobic interaction with the PAO, while the V323G mutant protein had only 10 interacting residues forming 10 hydrogen-bonded interactions, 1 hydrophobic interaction, and 1 salt bridge with the PAO (Additional file 2, S2-5, S2-6, S2-7). Both the R320P and V323G mutations were found to potentially decrease protein-ligand interactions.

### Stability analysis of OTC wild-type and mutant structures

**Fig. 5:**
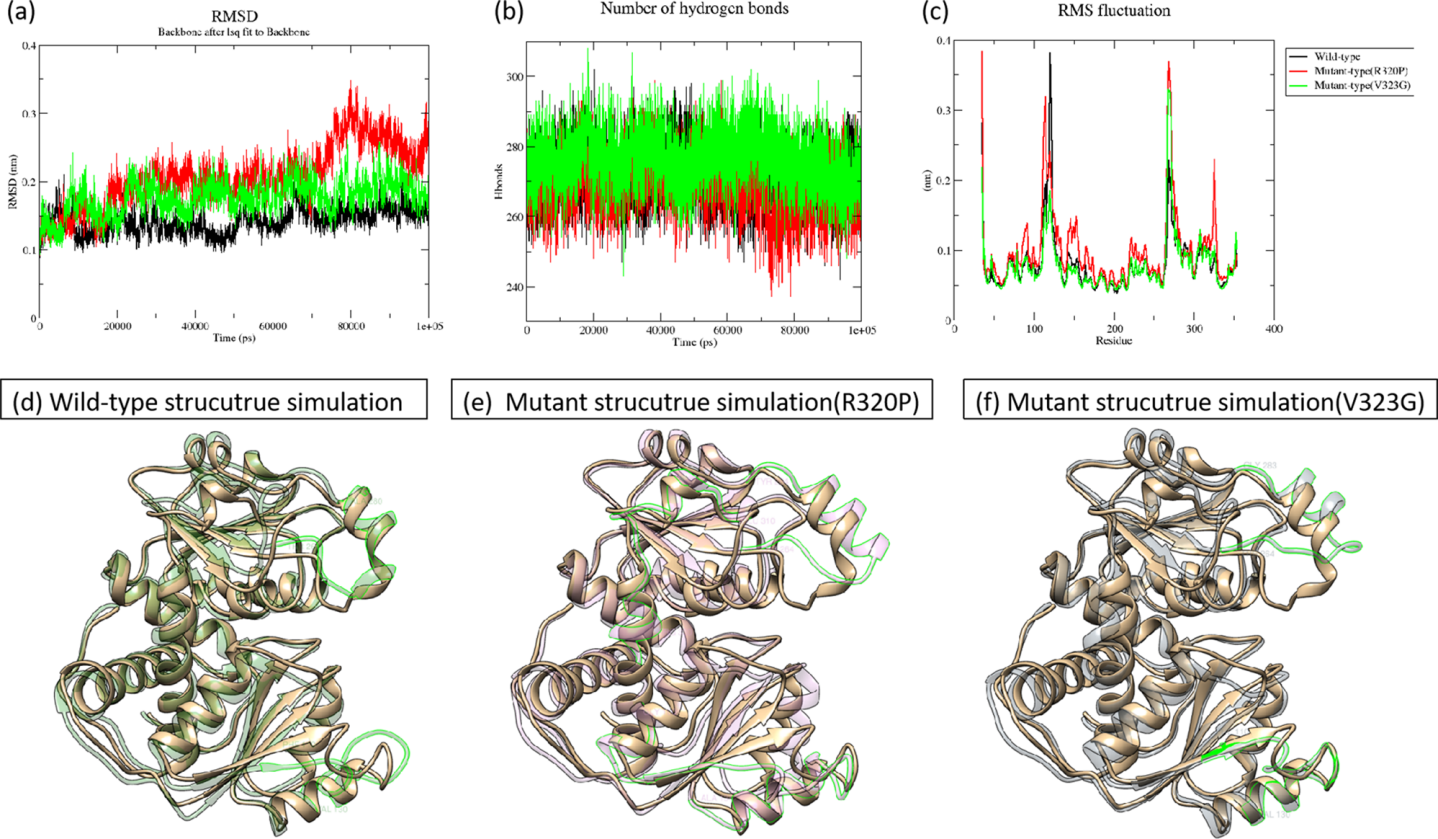
Analysis of molecular dynamics simulation results. (**a**) Showing the RMSD of the wild-type and mutant structures. (**b**) Showing the hydrogen bonding number of the wild-type and mutant structures. (**c**) Demonstration of RMSF of wild-type and mutant structures. black color in (**a**), (**b**) and (**c**) indicates wild-type structure, red color indicates R320P mutant structure and green color indicates V323G mutant structure. (**d**), (**e**) and (**f**) show the wild-type and mutant structures after 100 ns simulation. The gold structure is the wild-type initial structure, and the transparent green color of (**d**) Fig. indicates the wild-type protein structure after kinetic simulation, where the highlighted part (residues 110–130, 264–280) corresponds to the RMSF peak part. (**e**) Figure Transparent pink indicates the structure of R320P mutant after kinetic simulation, where the highlighted part (residues 100–135, 264–284, 310–330) corresponds to the RMSF peak part. (**f**) Figure Transparent gray indicates the V323G mutant structure after kinetic simulation, where the highlighted part (residues 110–130, 264–283) corresponds to the RMSF peak part

Molecular dynamics simulation techniques have the capability to replicate the dynamic behavior of protein molecules in aqueous solvents, thereby elucidating potential activities of protein molecules and offering valuable insights for both scientific investigation and clinical analysis. In this study, we employed the molecular dynamics simulation software GROMACS to conduct kinetic simulations on the wild-type and mutant structures of OTC proteins (Fig. 5).

The RMSD plot suggests that the protein system exhibits stability in molecular dynamics simulations. Conversely, analysis of the hydrogen bond number plot and RMSF plot indicates that the R320P mutant protein may induce local structural changes due to the mutation at this specific site. The elevated RMSF value implies that this region of the protein structure has undergone significant movement compared to its typical position over the course of the simulation. The root mean square fluctuations (RMSF) plots demonstrate notable peak fluctuations in the residue fragments of the R320P mutant protein structure (310–330) in comparison to the structure lacking the mutation at this site, suggesting that the mutation of this residue could potentially lead to decreased stability in this region. Subsequently, we generated the wild-type and mutant protein structures following molecular dynamics simulations, and identified the residues associated with the RMSF peaks (Fig. 5(d)(e)(f)). The software tool DoGSiteScorer was employed to forecast the area and volume of binding sites in both wild-type and mutant protein structures, as well as in each structure post-simulation (Table 4). The findings revealed a reduction in the area and volume of binding sites in the wild-type protein structure, suggesting a convergence of the two structural domains towards the protein core during the simulation. Conversely, the area and volume of binding sites in the two mutant protein structures exhibited an increase, with the most significant alterations observed in the binding sites of the R320P mutant protein structure.

**Table 4:**
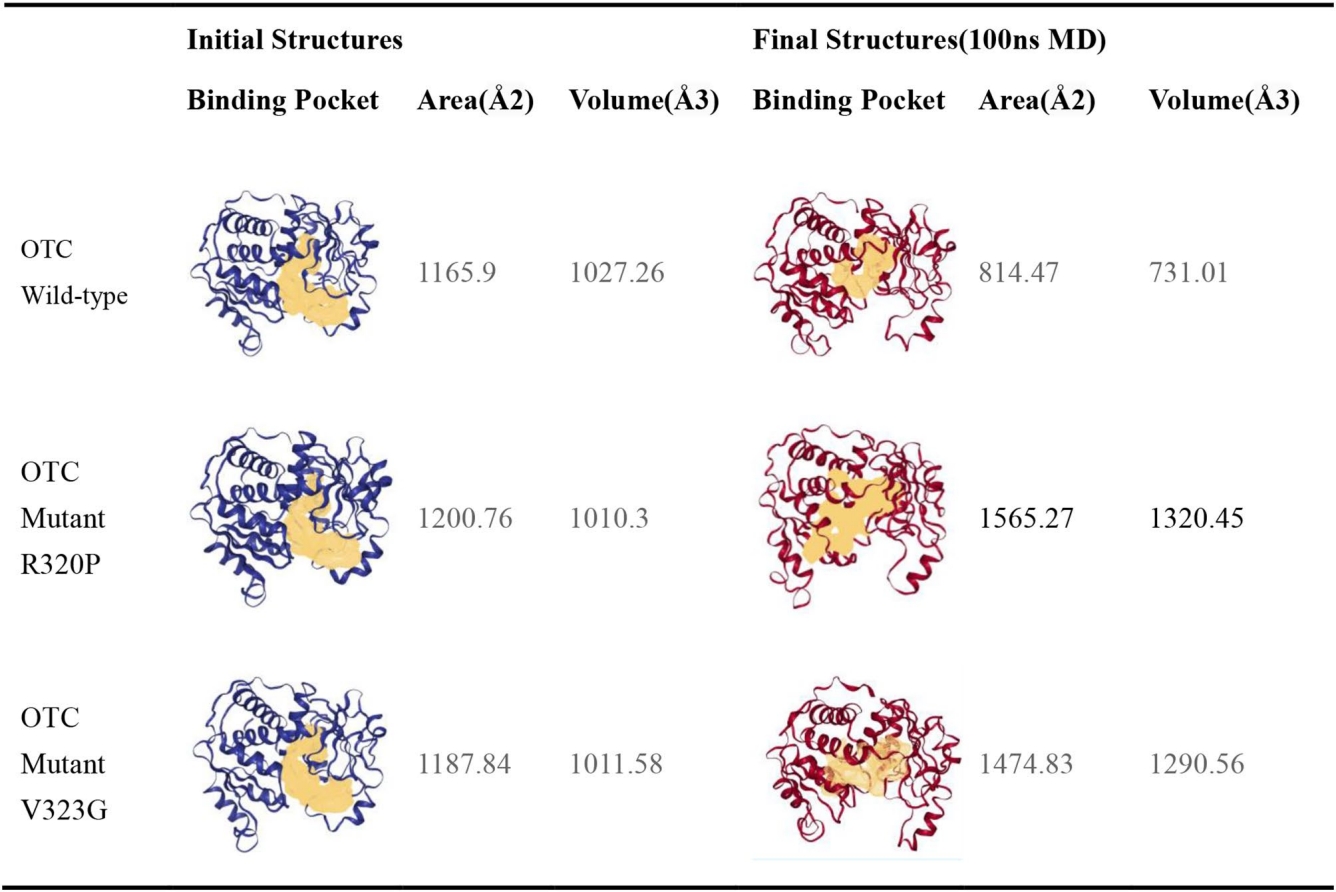
DoGSiteScorer was used to predict the area and volume of the binding sites

## Discussion

Twelve patients diagnosed with ornithine transcarbamylase deficiency (OTCD) were included in the study. The clinical and laboratory findings of these patients were assessed. The clinical manifestations of OTCD in this cohort were found to be non-specific, with 9 males presenting with early-onset symptoms, 2 females exhibiting a late-onset phenotype, and 1 male being asymptomatic, consistent with findings from previous studies [6, 25]. Furthermore, a high mortality rate was observed in this cohort, with 77.8% (7/9) of early-onset patients and 50% (1/2) of late-onset patients succumbing to the disease. Prompt diagnosis and emergency treatment are crucial in reducing the high fatality rate associated with OTCD. Genetic testing should be conducted promptly in high-risk patients for confirmed diagnosis.

The current diagnostic approach for OTCD involves a combination of clinical symptoms, specific biochemical detection, and genetic testing. Given the non-specific clinical manifestations of the disease, further investigation into the relationship between genotype and phenotype is warranted. Based on the analysis of references, the most prevalent OTC variants observed in the Chinese population were c.829 C > T(R277W) (6.53%), c.119G > A(R40H) (6.19%), and c.386G > A(R129H) (3.09%). This study aims to explore the genotype-phenotype relationships of these common mutations. For instance, the R277W variant, which occurs in CpG dinucleotides, has been documented in certain male hemizygotes exhibiting either asymptomatic or mild clinical presentations [26–28]. On the other hand, the R40H variant, affecting a CpG dinucleotide [29], is typically linked to a mild or late-onset phenotype [25, 30]. There are multiple CpG duplexes in the coding region of the *OTC* gene, including four TaqI restriction sites (TCGA) and two MspI restriction sites (CCGG), both the TaqI restriction site and the MspI restriction site on this CpG duplex have a high variant frequency [31], which is clearly associated with the high population detection rate at this locus. At present, there is a lack of comprehensive statistical data on the prevalence of the OTC (c.119G > A, R40H) variant and other related variants within the Chinese population. We are currently engaged in the collection and analysis of neonatal genetic screening data in South China, with the aim of determining the distribution of OTC gene variant sites and allele frequencies in the Chinese population.

In our research, patients exhibiting the same mutant allele displayed a range of clinical symptoms (Table 1 and Additional file 1), encompassing both early-onset and late-onset manifestations. Patients carrying the c.119G > A (R40H) variant exhibit highly heterogeneous clinical manifestations. While this variant is not present in the gnomAD database, it is classified as pathogenic or potentially pathogenic in the ClinVar database. Functional studies, such as those conducted by Augustin L [32], found that chinese hamster ovary cultured cells were transformed to continuously express wild-type and *OTC* gene (R40H mutation) causes a reduction of enzymatic activity to approximately 26 to 35% of wild type concomitant with a significant reduction in the amount of protein present. Therefore the allele frequency of c.119G > A (R40H) was not reported in Chinese OTCD population, but its pathogenicity is high according to previous studies. This variability in patient phenotype underscores the influence of not only allelic heterogeneity but also environmental factors such as infection, high-protein diet, and steroid therapy [3, 7, 8]. Additionally, our findings indicate that early-onset patients exhibit more pronounced blood ammonia and metabolic disturbances, leading to higher mortality rates, whereas late-onset patients experience more severe liver function impairment, potentially attributable to chronic liver injury resulting from infection or hyperammonemia [6].

Two novel variants of the OTC gene (c.968T > G V323G and c. 959G > C, R320P) were identified in patients with OTCD. The functional significance of these variants, classified as Variants of Uncertain Significance(VUS), remains unclear. Further biophysical analyses are necessary to elucidate and accurately predict the impact of these genetic mutations. In this study, we conducted in silico analysis of the two novel VUS variants of the OTC gene. Both MutPred2 and PolyPhen-2 algorithms predicted the two mutation sites as pathogenic. Additionally, PLIP analysis indicated that the mutations R320P and V323G may lead to a reduction in protein-ligand interactions. The findings from GROMACS molecular dynamics simulations suggest that the R320P mutant residue may induce instability in the 310–330 region of the structure, potentially disrupting the relative stability of the CP-binding/polar structural domain and the ORN-binding/equatorial structural domain. Therefore, in silico analysis offers compelling evidence supporting the pathogenic nature of the R320P and V323G mutations. Nevertheless, additional biochemical experiments are required to further investigate the correlation between genotype and phenotype, and additional biological experimental methodologies are necessary to validate the aforementioned phenomenon.

## Conclusion

In summary, this study examines the clinical presentations, biochemical manifestations, clinical outcomes, and mutation analysis of 12 Chinese patients with ornithine transcarbamylase deficiency (OTCD). Two novel mutations were identified, expanding the mutation spectrum of the *OTC* gene. Additionally, a comprehensive overview of the gene mutation spectrum in Chinese OTCD patients is presented, offering a foundation for genetic counseling. Through the utilization of in silico analysis, we have effectively elucidated the effects of two newly identified variations of uncertain significance (VUS) in the OTC gene on the functionality of the *OTC* protein within a three-dimensional structure. Furthermore, by examining the impact on domain function both pre- and post-mutation of amino acids, we can elucidate the role of amino acid substitutions in the development of OTCD.

## Electronic Supplementary Material

Below is the link to the electronic supplementary material.

Supplementary Material 1

Supplementary Material 2

## Data Availability

The data sets generated during and/or analyzed during the current study are available from the corresponding author on reasonable request.

## Abbreviations

OTCD: Ornithine transcarbamylase deficiency
OTC: Ornithine transcarbamylase
UCDs: Urea cycle disorders
PAO: N-(PHOSPHONOACETYL)-L-ORNITHINE
PDB: Protein Data Bank
PLIP: protein-ligand interaction profiler
VUS: Variants of Uncertain Significance
M: Male
F: Female
D: days
M: months
Y: years
EO: early-onset
LO: late-onset
AS: Asymptomatic
Lac: lactic acid
ALT: alanine aminotransferase
AST: glutamic-oxalacetic transaminase
TBIL: total bilirubin
