# Supplementary material for: Clinical and biochemical characteristics of patients with ornithine transcarbamylase deficiency and in silico analysis of OTC gene
Source: Orphanet J Rare Dis. 2025 Mar 18;20:131. doi: 10.1186/s13023-025-03624-4 (PMC11916849; doi:10.1186/s13023-025-03624-4)
Supplement: Supplementary file 1 — Supplementary Material 1 [file 13023_2025_3624_MOESM1_ESM.docx]

**Additional File 1**

OTC gene mutations, phenotypes, clinical and biochemistry features and clinical outcomes identified of OTCD Chinese patients in references.

| No. | Location | Nucleic acid | Amino acid | Clinical classification | References | Time | Phenotype | Sex | NH3 peak | Citrulline | Glutamine | Orotic acid | uracil | PH | Lac | BE | ALT | AST | TBIL | GnPn | GA | Outcomes | Clinical presentation |
| --- | --- | --- | --- | --- | --- | --- | --- | --- | --- | --- | --- | --- | --- | --- | --- | --- | --- | --- | --- | --- | --- | --- | --- |
|  |  |  |  |  |  |  |  |  |  |  |  |  |  |  |  |  |  |  |  |  |  |  |  |
| 1 | Intron 1 | c.2A>G | - | PAT | Liu Xiaojing(2013) | 6M | Late | M | 229.87 | 3 | NA | Elevated | Elevated | NA | 6.6 | NA | 740 | 427 | NA | G3P3 | NA | Alive | Cough, vomiting |
|  |  |  |  |  | Liu Ning(2020) | Neonate | Neonatal | M | NA | NA | NA | NA | NA | NA | NA | NA | NA | NA | NA | NA | NA | Alive | NA |
| 2 | Exon1 | c.3G>A | p.Met1Ile | PAT | Deyun Lu (2020) | 6Y | Late | F | 194 | 19.24 | 14.22 | 198.45 | 205.32 | NA | NA | NA | 509 | NA | NA | NA | NA | Alive | NA |
| 3 | Exon1 | c.67C>T | p.Arg23*  p.Arg23Ter | PAT  PAT | Deyun Lu (2020) | 2.5Y | Late | F | 190 | 15.75 | 13.5 | 202 | 67.5 | NA | NA | NA | 665 | NA | NA | NA | NA | Alive | NA |
|  |  |  |  |  | Wang Haijun(2018) | 2Y6M | Late | F | 208 | 14.627 | NA | 293.656 | 28.394 | 7.39 | NA | NA | 75 | 44 | NA | NA | NA | Alive | Sleepy, delayed motor development, muscular hypotonia |
|  |  |  |  |  | Wu Feifei(2018) | 2Y5M | Late | F | 361.2 | 10.264 | 42.02 | 56.8 | 133.9 | NA | 3.01 | NA | 313 | 64 | 22.7 | NA | NA | NA | Vomiting, and a disturbance of consciousness |
|  |  |  |  |  | Jiang Yongchao(2018) | 2Y8M | Late | F | 566 | Normal | Normal | Normal | Normal | NA | NA | NA | ＞7000 | ＞7000 | NA | NA | NA | Death | Vomiting, lethargy, and liver damage |
| 4 | Exon1 | c.77G>A | p.Arg26Gln | PAT | Yongxian Shao(2017) | 11D | Neonatal | M | 1365 | 3.5 | 2085 | NA | NA | NA | NA | NA | Normal | NA | NA | NA | NA | Death | Poor suck |
|  |  |  |  |  | Zhenzhu Zheng(2020) | 2D | Neonatal | M | 400 | 2.66 | NA | 132.92 | NA | NA | NA | NA | NA | NA | NA | NA | NA | Death | Severe jaundice, coma, respiratory depression |
|  |  |  |  |  | Huang Junzi(2024) | Neonate | Neonatal | M | 1145 | 8.5 | NA | 22.47 | NA | NA | NA | NA | NA | NA | NA | NA | 38^+1^ | Alive | NA |
| 5 | Intron 1 | c.77+1G>C | - | NR | Deyun Lu (2020) | 1D | Neonatal | F | 2000 | 2.88 | 21.31 | 282.12 | 34.56 | NA | NA | NA | ND | NA | NA | NA | NA | Death | NA |
| 6 | Intron 1 | c.78-2A>G | - | PAT | Deyun Lu (2020) | 3M | Late | M | 265 | 2.43 | 12.81 | 1.79 | 5.72 | NA | NA | NA | 105 | NA | NA | NA | NA | Death | NA |
|  |  |  |  |  | Wang Ming(2023) | 8M9D | Late | M | 155.4 | 6.01 | NA | 136 | 21.87 | NA | NA | NA | 187.7 | NA | NA | NA | NA | Alive | NA |
|  |  |  |  |  | Wang Ming(2023) | 1Y1M | Late | M | 150.9 | 4.567 | NA | 198.3 | 42.93 | NA | NA | NA | 51.8 | NA | NA | NA | NA | Alive | NA |
|  |  |  |  |  | Hua Xia(2017)  Zhao Jing(2019) | 7+M | Late | M | 168 | 4.833 | NA | 83.2 | 214.04 | NA | 3.9 | NA | 1612.7 | 1250.1 | NA | NA | NA | Alive | Sleepy，convulsion，vomiting |
| 7 | Exon2 | c.103insA | p.Val35Serfs*7 | NR | Yongxian Shao(2017)  Lu Zhikun(2024) | 2Y | Late | F | 580 | 26.2 | 939 | Elevated | Elevated | NA | NA | NA | NA | NA | NA | NA | NA | Death | Recurrent vomiting accompanied with lethargy |
| 8 | Exon 2 | c.116G>T | p.Gly39Val | NR | Zhenzhu Zheng(2020) | 1D | Neonatal | M | >280 | 2.55 | NA | NA | NA | NA | NA | NA | NA | NA | NA | NA | NA | Death | Jaundice, hypotonia, poor responses |
|  |  |  |  |  | Zhenzhu Zheng(2020) | 7M | Late | F | >280 | 19.46 | NA | 35.54 | NA | NA | NA | NA | 226 | 214 | NA | NA | NA | Alive | Hyperammonemia, liver dysfunction |
|  |  |  |  |  | Huang Junzi(2024) | Neonate | Neonatal | M | 982 | 4.92 | NA | 65.87 | NA | NA | NA | NA | NA | NA | NA | NA | 39^+2^ | Death | NA |
|  |  |  |  |  | Liu Ning(2020) | ＜3M | Late | M | NA | NA | NA | NA | NA | NA | NA | NA | NA | NA | NA | NA | NA | Alive | NA |
| 9 | Exon 2 | c.118C>T | p.Arg40Cys | PAT | Wang Haijun(2018) | 2Y10M | Late | F | 500 | 8.502 | NA | 151.02 | 5.117 | 7.414 | NA | NA | 2250 | 1909 | NA | NA | NA | Death | Sleepy，fidget，dystropy，vomiting，hypotonia |
| 10 | Exon 2 | c.119G>A | p.Arg40His | PAT | Yongxian Shao(2017) | 12Y | Late | M | 795 | 28.5 | 1216 | NA | NA | NA | NA | NA | Elevated | NA | NA | NA | NA | Death | Fever, seizure, coma |
|  |  |  |  |  | Qingnv Zhou(2020) | 7D | Neonatal | M | 310 | 5.953 | 14.047 | NA | 4.76 | 7.38 | 1.9 | -1.8 | 125 | NA | NA | NA | 40^+2^ | NA | Seizures,Somnolence,Decreased liver function,Acute encephalopathy,Feeding difficulties |
|  |  |  |  |  | Deyun Lu (2020) | 56Y | Late | M | 1152 | 8.83 | 5.84 | 827.28 | 28.69 | NA | NA | NA | 23 | NA | NA | NA | NA | Death | NA |
|  |  |  |  |  | Yuan Yuanhong(2019) | 14Y4M | Late | M | 446.7 | 12.41 | 85.59 | 143.7 | 24.7 | NA | 3.38 | NA | NA | NA | NA | G1P2 | 32 | Death | Convulsion, conscious disturbance ,vomiting |
|  |  |  |  |  | Yuan Yuanhong(2019) | 14Y4M | Asymptomatic | M | 45.2 | 11.6 | 25.96 | 0 | 3.3 | NA | NA | NA | NA | NA | NA | G1P1 | 32 | Normal | Fever, vomiting, and disturbance of consciousness |
|  |  |  |  |  | Wan Yahui(2019) | 15Y | Late | M | 566.2 | NA | NA | NA | NA | NA | 1.8 | NA | NA | NA | 85.7 | NA | NA | Alive | Asymptomatic |
|  |  |  |  |  | Jin Linmei(2020) | 19M | Late | M | 126 | 3.73 | NA | 395.1 | 44.9 | 7.49 | 7 | -7.6 | 247.2 | 265.1 | NA | NA | NA | Death | Convulsion, conscious disturbance ,vomiting |
|  |  |  |  |  | Wang Yahui(2021) | 13Y3M | Late | M | 566.2 | NA | NA | NA | NA | NA | 7.8 | NA | Normal | Normal | 85.7 | NA | NA | Alive | Fever, conscious disturbance ,vomiting |
|  |  |  |  |  | Yan Beibei(2021) | 4Y | Late | M | 1263 | 3.83 | NA | 256.1 | NA | 7.46 | 4.8 | -5.3 | 64 | 82 | NA | G1P1 | NA | Death | Fever, convulsions, coma, and vomiting |
|  |  |  |  |  | Zhang Sufang(2023) | 4Y | Late | M | 1263 | 3.83 | NA | NA | NA | NA | NA | NA | NA | NA | NA | NA | NA | Alive | Vomiting, drowsiness, and coma |
|  |  |  |  |  | Xin Jin(2022) | 11Y | Late | NA | ＞500 | Normal | NA | Elevated | Elevated | NA | 4.47 | NA | 73 | NA | NA | NA | NA | Alive | Unexplained headaches,vomiting,consciousness disturbance |
|  |  |  |  |  | Luo Zhiqiang(2023) | 11Y8M | Late | M | 563.6 | 14.7 | NA | 123.2 | 29.4 | 6.9 | NA | NA | Normal | Normal | NA | NA | NA | Alive | Headache, vomiting, and disturbance of consciousness |
|  |  |  |  |  | Xie Lei(2023) | 15Y3M | Late | M | 179.9 | Normal | Normal | Elevated | Elevated | NA | NA | NA | NA | NA | 32.75 | G3P3 | NA | NA | Disorder of consciousness, vomiting |
|  |  |  |  |  | Xie Lei(2023) | 4Y8M | Late | M | 101.6 | NA | NA | NA | NA | 2.84 | NA | NA | 75 | NA | NA | G1P1 | NA | NA | Titching, abdominal pain, vomiting |
|  |  |  |  |  | Zhao Jing(2019)  Wang Ming(2023) | 13Y3M | Late | M | 129 | 7.079 | NA | 1.4 | 34.2 | NA | NA | NA | 2242.6 | 889.4 | NA | NA | NA | Alive | Disorder of consciousness, vomiting, panic, irritability, and involuntary movement |
|  |  |  |  |  | Sun Yuan(2019) | 17Y | Late | M | 2998 | Normal | Normal | Normal | Normal | 7.51 | NA | NA | 263 | 95 | NA | NA | NA | Death | Episode dizziness, vomiting, convulsions |
|  |  |  |  |  | Sun Yuan(2019) | 15Y | Late | M | 600 | Normal | Normal | Normal | Normal | NA | NA | NA | 601 | 298 | NA | NA | NA | Alive | Epodic dizziness, vomiting, lethargy |
|  |  |  |  |  | Wu Jie(2022) | 55Y | Late | M | 4679 | Normal | NA | Elevated | Elevated | NA | NA | NA | NA | NA | NA | NA | NA | Death | Fever, vomiting, disturbance of consciousness, and seizures |
| 11 | Exon 2 | c.122A>C | p.Asp41Ala | NR | Deyun Lu (2020) | 1.8Y | Late | M | 180 | 3.07 | 27.2 | ND | 17.93 | NA | NA | NA | 42 | NA | NA | NA | NA | Alive | NA |
| 12 | Exon 2 | c.124_126del | p.Leu42del | NR | Deyun Lu (2020) | 1.1Y | Late | F | 300 | 3.17 | 14.11 | 77.65 | 29.97 | NA | NA | NA | 303 | NA | NA | NA | NA | Alive | NA |
| 13 | Exon 2 | c.125T>C | p.Leu42Pro | NR | Jiang Fangfang(2015) | 1Y9M | Late | F | ＞1000 | 16.76 | NA | NA | 163.43 | NA | NA | NA | 1677.5 | 853.2 | 10.2 | NA | NA | Alive | Vomiting, and had a poor mental response |
| 14 | Exon 2 | c.140dupA | p.Asn47Lysfs*8 | NR | Zhou duo(2023) | 2Y9M | Late | F | 500 | Normal | Normal | Elevated | Elevated | NA | NA | NA | 2083 | 1246 | NA | NA | NA | Alive | Poor gastric appetite, abdominal pain, and vomiting |
| 15 | Exon 2 | c.140del | p.Asn47Thrfs*17 | VUS | Deyun Lu (2020) | 5.6Y | Late | F | 500 | 12.6 | 11.65 | 189.2 | 50.04 | NA | NA | NA | 759 | NA | NA | NA | NA | Death | NA |
| 16 | Exon 2 | c.148 G ＞ T | p． G50X | NR | Tang Jianping(2017) | 10D | Neonatal | M | NA | NA | NA | Elevated | Elevated | NA | Elevated | NA | NA | NA | NA | NA | NA | NA | High lactic acid, high blood ammonia, jaundice, and convulsions |
| 17 | Exon 2 | c.167T>A | - | NR | Huang Junzi(2024) | Neonate | Neonatal | M | 1020 | 3.85 | NA | 35.61 | NA | NA | NA | NA | NA | NA | NA | NA | 40^+2^ | Alive | NA |
| 18 | Exon 2 | c.174G>A | p.Trp58* | PAT | Deyun Lu (2020) | 2Y | Late | F | ND | 14.07 | 18.96 | 141.39 | 35.73 | NA | NA | NA | 40 | NA | NA | NA | NA | Withdraw | NA |
| 19 | Exon 2 | c.176T>C | p.Leu59Pro | NR | Qingnv Zhou(2020) | 2D | Neonatal | M | 1367 | 6.261 | 97.444 | 641.56 | 46.34 | 7.4 | 8.2 | 0.1 | 25 | NA | NA | NA | 39^+6^ | NA | Seizures,Somnolence,Acute encephalopathy,Coma,Fever |
|  |  |  |  |  | Fu Dalin(2019) | 6D | Neonatal | M | ＞500 | NA | NA | NA | NA | 7.67 | 5.7 | 0.8 | NA | NA | NA | G4P1 | 37^+1^ | Death | Stress of breath, poor response, fever, and convulsions |
| 20 | Exon 2 | c.177delA | p.S 60 QfsTer4 | NR | Chu Xiaoyun(2023) | 1D | Neonatal | M | 1574 | 4.9 | 2 776 . 5 | 110.8 | NA | NA | 13.7 | NA | NA | NA | NA | NA | 39^+1^ | Death | Anhelation，Eating difficult |
| 21 | Exon 2-3 | c.207-226del20 | - | NR | Deyun Lu (2020) | 1.4Y | Late | F | 197 | 8.82 | 49.73 | 691.94 | 559.21 | NA | NA | NA | 1639 | NA | NA | NA | NA | Alive | NA |
| 22 | Exon 3 | c.231G>C | p.Leu77Phe | NR | Yongxian Shao(2017) Min-Zhi Peng(2020) | 15Y | Late | M | 156 | 14.5 | 1358 | NA | NA | NA | NA | NA | Normal | NA | NA | NA | NA | Alive | Recurrent vomiting |
| 23 | Exon 3 | c.234A>G | p.Gln78Gln | VUS | Wang Ming(2023) | 1Y1M | Late | F | 327.1 | 7.88 | NA | 50.34 | 104 | NA | NA | NA | 74.3 | NA | NA | NA | NA | Alive | NA |
| 24 | Exon 3 | c. 241T>C | p. S81P | NR | Yang Fan(2024) | 6Y6M | Late | M | 327 | 6.05 | NA | NA | 14.4 | NA | Normal | NA | 140 | 160 | NA | G6P3 | NA | Alive | Vomiting, convulsive seizures, drowsiness, and unconsciousness |
| 25 | Exon 3 | c.248G>A | p.Gly83Asp | PAT | Zhu Zhijun(2015) | 27M | Late | F | 270 | NA | NA | NA | NA | NA | NA | NA | NA | NA | NA | NA | NA | Alive | Fgety, mental abnormalities |
| 26 | Exon 3 | c.270T>G | p.Ser90Arg | PAT | Min-Zhi Peng(2020) | 3Y5M | Late | F | NA | NA | NA | NA | NA | NA | NA | NA | NA | NA | NA | NA | NA | Alive | Recurrent vomiting, irritability |
|  |  |  |  |  | Yongxian Shao(2017) | 4Y | Late | F | 282 | 21.7 | 809 | NA | NA | NA | NA | NA | Elevated | NA | NA | NA | NA | Alive | Vomiting |
| 27 | Exon 3 | c.274C>T | p.Arg92* | PAT | Fang Weiyuan(2023) | 4Y9M | Late | F | 41 | 35.63 | NA | 0 | 0 | Normal | Normal | Normal | 218 | 167 | 10.4 | G3P2 | NA | Alive | Dysfunction of blood coagulation |
|  |  |  |  |  | Yan Beibei(2021) | 3D | Neonatal | M | 3234 | 19.91 | NA | 155 | NA | 7.23 | 7.5 | -5 | 14 | 51 | NA | G7P5 | 39 | Death | Convulsive, poor response, blue, coma |
| 28 | Exon 3 | c.275G>A | p.Arg92Gln | PAT | Deyun Lu (2020) | 1.5Y | Late | F | 103 | 8.81 | 9.87 | 48.92 | 71.86 | NA | NA | NA | 208 | NA | NA | NA | NA | Death | NA |
|  |  |  |  |  | Zhu Zhijun(2015) | 17M | Late | F | 470 | NA | NA | NA | NA | NA | NA | NA | NA | NA | NA | NA | NA | Alive | Fgety, vomiting, disturbance of consciousness |
|  |  |  |  |  | Gao Ruiwei(2023) | 15H | Neonatal | M | 1168 | 2.83 | NA | Elevated | NA | 7.068 | 8.2 | -10.7 | NA | NA | NA | G1P1 | 36 | NA | Dynobreathing, convulsions and poor response |
|  |  |  |  |  | Wang Haijun(2018) | 2Y2M | Late | F | 2243 | 6.939 | NA | 58.453 | 28.259 | 7.312 | NA | NA | 385 | 422 | NA | NA | NA | Death | Sleepy, talk nonsense, cough, abdominal pain |
| 29 | Intron 3 | c.298+2T>G | - | PAT | Deyun Lu (2020) | 3.5Y | Late | F | 234 | 9.53 | 17.25 | 6.75 | 234 | NA | NA | NA | 687 | NA | NA | NA | NA | Alive | NA |
| 30 | Intron 3 | c.298+5G>C | - | Benign | Deyun Lu (2020) | 1.3Y | Late | F | 189 | 8.21 | 26.24 | 227.12 | 441.98 | NA | NA | NA | 139 | NA | NA | NA | NA | Alive | NA |
|  |  |  |  |  | Wang Ming(2023) | 2M16D | Late | M | 72.5 | 24.14 | NA | NA | 0.72 | NA | NA | NA | 26.6 | NA | NA | NA | NA | Death | NA |
| 31 | Exon 4 | c.317G>T | p.Gly106Val | PAT | Deyun Lu (2020) | 2.3Y | Late | F | 1300 | 9.7 | 12.78 | 43.5 | 251 | NA | NA | NA | NA | NA | NA | NA | NA | Alive | NA |
| 32 | Exon 4 | c.356G>A | p.Gly119Asp | Likely PAT | Rixiati(2024) | 3Y11M | Late | M | 53 | Normal | NA | NA | NA | NA | 3.32 | NA | 498 | 607 | NA | G3P2 | NA | NA | Abnormal liver function, mental retardation, hyperactivity, abnormal mood, and aggressive behavior |
| 33 | Exon 4 | c.364G>C | p.e122q | Likely PAT | Wang Jingwen(2023) | 8M | Late | M | 238 | 3.95 | NA | 123.48 | 106.77 | NA | 6.4 | NA | 197 | 247 | NA | NA | NA | Alive | Wagging spirit, vomiting |
| 34 | Exon 4 | c.365A>G | p.Glu122Gly | Likely PAT | Gao Hua(2003) | 3Y6M | Late | F | 138 | NA | NA | NA | NA | NA | NA | NA | NA | NA | NA | NA | NA | Death | Vomiting, coma |
| 35 | Exon 4 | c.367-368delAG | p.L124Hfs*9 | PAT | Wang Ming(2023) | 6Y1M | Late | F | 314.3 | 11 | NA | 288.54 | 6.19 | NA | NA | NA | 1141 | NA | NA | NA | NA | Alive | NA |
| 36 | Exon 4 | c.368delG | - | NR | Min-Zhi Peng(2020) | 1Y4M | Late | F | NA | NA | NA | NA | NA | NA | NA | NA | NA | NA | NA | NA | NA | Alive | Recurrent vomiting, irritability, light coma |
| 37 | Exon 4 | c.386G>A | p.Arg129His | PAT | Yongxian Shao(2017)  Min-Zhi Peng(2020) | 9M | Late | M | 170 | 7.1 | 1552 | NA | NA | NA | NA | NA | Elevated | NA | NA | NA | NA | Alive | Light Coma, partial seizure, psychomotor retardation |
|  |  |  |  |  | Yongxian Shao(2017)  Min-Zhi Peng(2020) | 1Y | Late | M | 336 | 5 | 995 | NA | NA | NA | NA | NA | Elevated | NA | NA | NA | NA | Alive | Generalized seizures, light coma, vomiting |
|  |  |  |  |  | Yongxian Shao(2017)  Min-Zhi Peng(2020) | 2Y | Late | M | 160 | 4.6 | 1051 | NA | NA | NA | NA | NA | Normal | NA | NA | NA | NA | Alive | Vomiting, irritability |
|  |  |  |  |  | Deyun Lu (2020) | 7D | Neonatal | M | 300 | 1.71 | 3.25 | 425.42 | 8.6 | NA | NA | NA | 32 | NA | NA | NA | NA | Alive | NA |
|  |  |  |  |  | Wang Yanyun(2017) | 10M | Late | F | ＞200 | 3.93 | Normal | Elevated | Elevated | NA | NA | NA | NA | NA | NA | NA | NA | Death | Vomiting, convulsions, and drowsiness |
|  |  |  |  |  | Wenjia Tong(2018) | 1Y | Late | F | NA | Reduce | NA | NA | NA | NA | NA | NA | NA | NA | NA | NA | NA | NA | Vomiting, Confusion, Hyperammonemia, Low plasma citrulline, Hypoargininemia, Brain hernia, Central cardiovascular failure, Respiratory difculties |
|  |  |  |  |  | Zhou Duo(2023) | Neonate | Neonatal | M | 360 | 4.92 | NA | NA | Elevated | NA | 3.3 | NA | 698 | 302 | NA | NA | NA | Withdraw | Hyperammonemia, with impaired liver function |
|  |  |  |  |  | Liu Li(2022) | Neonate | Neonatal | NA | NA | 3.76 | NA | NA | NA | NA | NA | NA | NA | NA | NA | NA | NA | Death | Newborn screening has confirmed the diagnosis |
|  |  |  |  |  | Tong Wenjia(2015) | 9Y | Late | M | 2500 | 6.48 | 44.88 | 172.8 | 27.8 | 7.428 | 3.107 | -8.12 | 32 | 45 | NA | NA | NA | Death | Vomiting, and resulting in confusion |
| 38 | Exon 4 | c.386G>T | p.Arg129Leu | PAT | Deyun Lu (2020) | 8M | Late | M | 300 | ND | ND | ND | ND | NA | NA | NA | ND | NA | NA | NA | NA | Alive | NA |
| 39 | Intron 4 | c.386+1G>A  IVS4+1G>A | - | PAT | Yongxian Shao(2017)  Min-Zhi Peng(2020) | 1Y4M | Late | M | 690 | 12.7 | 1019 | NA | NA | NA | NA | NA | Elevated | NA | NA | NA | NA | Death | Recurrent vomiting accompanied with lethargy, severe coma |
| 40 | Intron 4 | c. 387 - 1 G>T | - | NR | Chu Xiaoyun(2023) | 1D | Neonatal | M | 1700 | 4.6 | 3841.2 | 106.4 | NA | NA | 14 | NA | NA | NA | NA | NA | 38 | Death | Eating difficult |
| 41 | Exon 5 | c.406G>T | p.D136Y | Likely PAT | Wu Feifei(2018) | 6Y7M | Late | F | 469.5 | 17.98 | 57.577 | 9.2 | 16.5 | NA | 3.93 | NA | 41 | 64 | 15.7 | NA | NA | Death | Poor spirits, vomiting |
| 42 | Exon 5 | c.410C>A | p.Ala137E | VUS | Song Haijiang(2021) | 2Y10M | Late | F | 500 | NA | NA | NA | NA | NA | NA | NA | 679 | NA | 12.8 | NA | NA | Alive | Repeated vomiting, easy irritability |
| 43 | Exon 5 | c.416T>G | - | PAT | Wang Haijun(2018) | 2Y10M | Late | M | 524 | 4.674 | NA | 1182.548 | 249.891 | 7.242 | NA | NA | 80 | 44 | NA | NA | NA | Alive | Repeated vomiting, irritability, convulsions |
| 44 | Exon 5 | c.421C>T | p.Arg141* | PAT | Yongxian Shao(2017) | 1Y | Late | F | 575 | 9.8 | 494 | NA | NA | NA | NA | NA | Elevated | NA | NA | NA | NA | Death | Vomiting |
|  |  |  |  |  | Min-Zhi Peng(2020) | 2Y | Late | F | NA | NA | NA | NA | NA | NA | NA | NA | NA | NA | NA | NA | NA | Death | Recurrent vomiting accompanied with lethargy |
|  |  |  |  |  | Xie Lei(2023) | 2Y6M | Late | F | 558.1 | Reduce | NA | Reduce | Elevated | NA | 2.6 | NA | 330 | 285 | NA | G3P3 | NA | NA | Mental abnormalities, vomiting, abnormal liver function, and convulsions |
| 45 | Exon 5 | c.421C>G | p.Arg141Gly | PAT | Deyun Lu (2020) | 4M | Late | F | 59 | 6.61 | ND | 224.31 | ND | NA | NA | NA | 28 | NA | NA | NA | NA | Alive | NA |
| 46 | Exon 5 | c.422G>A | p.Arg141Gln | PAT | Deyun Lu (2020) | 2.3Y | Late | F | 120 | 11.84 | 4.93 | 43.83 | 131.88 | NA | NA | NA | 215 | NA | NA | NA | NA | Death | NA |
|  |  |  |  |  | Wang Ming(2023) | 3Y10M | Late | F | 103.1 | 14.2 | NA | 60.3 | 87.31 | NA | NA | NA | 1401 | NA | NA | NA | NA | Alive | NA |
|  |  |  |  |  | Wang Ming(2023) | 1Y11M | Late | F | 409 | 18.11 | NA | 267.87 | 18.86 | NA | NA | NA | 349 | NA | NA | NA | NA | Alive | NA |
|  |  |  |  |  | Zhang Sufang(2023) | 2Y5M | Late | F | 419 | NA | NA | Elevated | Elevated | NA | NA | NA | NA | NA | NA | NA | NA | Alive | Convulsive, and unconsciousness |
|  |  |  |  |  | Zhou Duo(2023) | 2Y5M | Late | F | 419 | Normal | NA | Elevated | Elevated | NA | NA | NA | 472 | 305 | NA | NA | NA | Alive | Delirium, repeated convulsions |
| 47 | Exon 5 | c.475A>G | p.Ile159Val | VUS | Wang Ming(2023) | 4M29D | Late | M | 206 | 22.76 | NA | 1.31 | BA | NA | NA | NA | 517 | NA | NA | NA | NA | Death | NA |
| 48 | Exon 5 | c.482A>G | p.Asn161Ser | PAT | Yongxian Shao(2017) | 1Y | Late | F | 564 | 14 | 1199 | NA | NA | NA | NA | NA | Elevated | NA | NA | NA | NA | Death | Vomiting |
|  |  |  |  |  | Deyun Lu (2020) | 3D | Neonatal | M | 346 | ND | ND | ND | ND | NA | NA | NA | 18 | NA | NA | NA | NA | Death | NA |
|  |  |  |  |  | Wang Haijun(2018) | 6Y10M | Late | F | 349 | 22.4 | NA | 6.343 | 4.9 | 7.338 | NA | NA | 36 | 48 | NA | NA | NA | Alive | Dizziness, vomiting, and intellectual backwardness |
|  |  |  |  |  | Min-Zhi Peng(2020) | 1Y7M | Late | F | NA | NA | NA | NA | NA | NA | NA | NA | NA | NA | NA | NA | NA | Death | Recurrent vomiting accompanied with lethargy, irritability, partial seizure, avoidance of meat |
|  |  |  |  |  | Jin Di(2021) | 2D | Neonatal | F | 385 | NA | NA | NA | NA | 7.56 | 7.4 | -0.4 | NA | NA | NA | G2P2 | 38^+5^ | Death | Feeding difficulties |
|  |  |  |  |  | Jin Di(2021) | 3D | Neonatal | M | 653 | Reduce | NA | Elevated | NA | 7.18 | 10.3 | -6 | NA | NA | NA | G1P1 | 35^+2^ | Death | Feeding difficulties, convulsions |
|  |  |  |  |  | Liu Ning(2020) | 1Y | Late | F | NA | NA | NA | NA | NA | NA | NA | NA | NA | NA | NA | NA | NA | Alive | NA |
| 49 | Exon 5 | c.504T>A | p.His168Gln | PAT | Min-Zhi Peng(2020) | 1Y2M | Late | M | NA | NA | NA | NA | NA | NA | NA | NA | NA | NA | NA | NA | NA | Alive | Lethargy |
| 50 | Exon 5 | c.506C>T | p.Pro169Leu | PAT | Hua Xia(2016)  Wang Ming(2023)  Zhao Jing(2019) | 4Y8M | Late | M | 1714 | 10 | NA | 0 | 25.34 | NA | 4 | NA | 467.3 | 2134.3 | NA | NA | NA | Death | Comas, convulsions, vomiting, nonsense, hallucinations, involuntary movement |
|  |  |  |  |  | Li Jun（2017） | 4Y | Late | M | 1126 | 9.917 | 1.832 | 0 | 25.34 | NA | NA | NA | 170.4 | 86.8 | NA | NA | NA | Death | Vomiting, talking nonsense, convulsions, and coma |
| 51 | Exon 5 | c.512A>G | p.Q171R | PAT | Cai(2021) | 2D | Neonatal | M | 1250 | 2.91 | NA | 46.681 | 1.041 | 7.15 | 15.5 | -10.1 | NA | NA | NA | G1P1 | NA | Death | Coma, vomiting, and drowsiness |
| 52 | Exon 5 | c.514-35C>G | - | PAT | Zhou Duo(2023) | neonate | Late | M | 50 | 3.47 | NA | Normal | Normal | NA | Normal | NA | Normal | Normal | NA | NA | NA | NA | Repeated convulsions, large liver, epilepsy |
| 53 | Exon 5 | c.516C>G | p.Ile172Met | PAT | Yongxian Shao(2017),Min-Zhi Peng(2020) | 20D | Neonatal | M | 500 | 10.6 | 1032 | NA | NA | NA | NA | NA | Elevated | NA | NA | NA | NA | Alive | Light coma |
|  |  |  |  |  | Mo Weiqian(2011) | 13D | Neonatal | M | 500 | 1.12 | 14.85 | Elevated | Elevated | Normal | Normal | Normal | NA | NA | 105.7 | G1P1 | 38 | NA | Somnolence |
| 54 | Exon 5 | c.527A>G | p.Tyr176Cys | PAT | Min-Zhi Peng(2020) | 13Y4M | Late | M | NA | NA | NA | NA | NA | NA | NA | NA | NA | NA | NA | NA | NA | Alive | Recurrent vomiting accompanied with lethargy, generalized seizure, light coma |
| 55 | Exon 5 | c.533C>T | p.Thr178Met | PAT | Yu Mei(2019) | Neonate | Neonatal | M | NA | Reduce | NA | NA | NA | NA | NA | NA | NA | NA | NA | NA | NA | Death | Tolerance, drowsiness, low response, convulsions, coma |
| 56 | Exon 5 | c.540G>C | p.Gln180His | PAT | Deyun Lu (2020) | 2Y | Late | F | 256 | 7.68 | 32.68 | ND | ND | NA | NA | NA | ND | NA | NA | NA | NA | Alive | NA |
|  |  |  |  |  | Qingnv Zhou(2020) | 7D | Neonatal | M | 290 | 6.112 | 58.012 | 357.21 | 35.12 | 7.42 | 6.4 | -4.8 | 34 | NA | NA | NA | 38^+2^ | NA | Seizures,Somnolence,Acute encephalopathy, Coma, Fever, Feeding difficulties |
| 57 | Exon 5 | c.540G>T | pGln180His | NR | Qiu Jinshou(2023) | 7D | Neonatal | M | 845.6 | NA | NA | NA | NA | 7.24 | 11.1 | 2.2 | 27.4 | NA | 313.8 | G2P2 | 38^+3^ | Alive | Unrie, poor reaction, moaning |
| 58 | Exon 5 | del | - | NR | Huang Junzi(2024) | Neonate | Neonatal | M | 980 | 2.55 | NA | 307.09 | NA | NA | NA | NA | NA | NA | NA | NA | 39^+3^ | NA | NA |
| 59 | Exon 5 | insSINE？ | - | NR | Wu Feifei(2018) | 3D | Neonatal | M | 2516.1 | 3.07 | 106.12 | 0 | 5.2 | NA | 5.42 | NA | 42 | 243 | 180.1 | NA | NA | Death | Convulsion |
| 60 | Intron 5 | c.540+2T>C | - | PAT | Min-Zhi Peng(2020) | 1Y | Late | F | NA | NA | NA | NA | NA | NA | NA | NA | NA | NA | NA | NA | NA | Alive | Recurrent vomiting accompanied with lethargy |
| 61 | Intron 5 | c.540+265G>A | - | PAT | Deyun Lu (2020) | 1Y | Late | F | 480 | 4.21 | 21.26 | 4.53 | ND | NA | NA | NA | 97 | NA | NA | NA | NA | Alive | NA |
| 62 | Intron 5 | c.542+134:G>G/A | - | NR | Sun Weihua（2011） | 3D | Neonatal | M | 235 | 1.06 | NA | 124 | NA | 7.17 | 5.3 | NA | 52 | 32 | 312.4 | G1P1 | 41^+2^ | Death | Poor response, lethargy, dyspnea, and convulsions |
| 63 | Exon 6 | c.548A>G | p.Tyr183Cys | PAT | Zhao Jing(2019)  Wang Ming(2023) | 1Y6M | Late | F | 361 | 5.47 | NA | 0 | 44.3 | NA | NA | NA | 644.8 | 463.1 | NA | NA | NA | Alive | Convulsive, vomiting, irritability and irritability |
|  |  |  |  |  | WangYan(2014) | 1D | Neonatal | M | 1000 | 1.81 | 275.82 | Elevated | Elevated | 7.18 | NA | 4.5 | NA | NA | NA | G5P4 | NA | NA | Titching, difficulty breathing |
| 64 | Exon 6 | c.548A>T | - | NR | Jiandi Gao(2015) | 2.5Y | Late | F | 420 | 13.17 | 22.37 | 315.64 | 942.4 | NA | 2.7 | NA | 1328 | 1014 | NA | NA | NA | Death | Vomiting, drowsiness.irascibility |
| 65 | Exon 6 | c.552insGAAC | p.Ser185Efs*41 | NR | Deyun Lu (2020) | 2.4Y | Late | F | 385 | 13.44 | 61.66 | 673.28 | 161.53 | NA | NA | NA | 380 | NA | NA | NA | NA | Death | NA |
|  |  |  |  |  | Jiandi Gao(2015) | 2.5Y | Late | F | 420 | 13.17 | 22.37 | 315.64 | 942.4 | NA | 2.7 | NA | 1328 | 1014 | NA | NA | NA | Death | Vomiting, drowsiness.irascibility |
| 66 | Exon 6 | c.562G>C | p.Gly188Arg | PAT | Deyun Lu (2020) | 2Y | Late | M | 218 | 5.74 | 10.83 | 65.68 | 29.17 | NA | NA | NA | NA | ND | NA | NA | NA | Withdraw | NA |
| 67 | Exon 6 | c.579G>A | p.Trp193* | NR | Deyun Lu (2020) | 20D | Neonatal | M | ND | ND | ND | ND | ND | NA | NA | NA | ND | NA | NA | NA | NA | Death | NA |
| 68 | Exon 6 | c.583G>A | p.Gly195Arg | PAT | Lixia Li（2020） | 26Y | Late | F | 499 | NA | NA | NA | NA | NA | NA | NA | 121 | 109 | NA | NA | NA | Alive | Vomiting, limb numbness, poor speech, drowsiness |
|  |  |  |  |  | Deyun Lu (2020) | 3D | Neonatal | M | 637 | 3.09 | 33.54 | 231.2 | 151.2 | NA | NA | NA | ND | NA | NA | NA | NA | Death | NA |
|  |  |  |  |  | Deyun Lu (2020) | 6M | Late | F | 387 | 12.68 | 20.81 | 1435.31 | 153.89 | NA | NA | NA | 214 | NA | NA | NA | NA | Alive | NA |
|  |  |  |  |  | Deyun Lu (2020) | 29Y | Late | F | 421 | 8.41 | 18.16 | 38.75 | 51.55 | NA | NA | NA | ND | NA | NA | NA | NA | Death | NA |
|  |  |  |  |  | Yang Lingyun(2015)  Wang Li(2015)  Qingnv Zhou(2020) | 1H | Neonatal | M | 1020 | 27.4 | NA | 1372.7 | 67.1 | 7.21 | 8 | -10.1 | NA | NA | NA | G1P1 | 32 | Death | Full body blue, irregular breathing |
|  |  |  |  |  | Liu Ning(2020) | neonate | Neonatal | M | NA | NA | NA | NA | NA | NA | NA | NA | NA | NA | NA | NA | NA | Death | NA |
| 69 | Exon 6 | c.586G>A | p.Asp196Asn | PAT | Yongxian Shao(2017) | 10M | Late | M | 177 | 9.5 | 1585 | NA | NA | NA | NA | NA | Normal | NA | NA | NA | NA | Alive | Vomiting, irritability |
|  |  |  |  |  | Yongxian Shao(2017) | 6Y | Late | M | 96 | 11.3 | 1051 | NA | NA | NA | NA | NA | Elevated | NA | NA | NA | NA | Alive | Vomiting |
|  |  |  |  |  | Min-Zhi Peng(2020) | 10M | Late | M | NA | NA | NA | NA | NA | NA | NA | NA | NA | NA | NA | NA | NA | Alive | Recurrent vomiting, irritability |
|  |  |  |  |  | Min-Zhi Peng(2020) | 10M | Late | M | NA | NA | NA | NA | NA | NA | NA | NA | NA | NA | NA | NA | NA | Alive | Vomiting |
|  |  |  |  |  | Chen Zhanling(2014) | 1Y8M | Late | M | 612 | Elevated | NA | NA | NA | NA | 5.1 | NA | 49 | 81 | NA | NA | NA | Death | Infectious diarrhea, and hyperammonemia |
|  |  |  |  |  | Chen Zhanling(2014) | 11M | Late | M | 129 | 3.4 | NA | Normal | Elevated | NA | NA | NA | NA | NA | NA | NA | NA | Alive | Cerebral palsy and motor dysplasia |
| 70 | Exon 6 | c.591C>A* | p.Leu201Met | NR | Yongxian Shao(2017) | 1Y | Late | F | 473 | 13.6 | 997 | NA | NA | NA | NA | NA | Elevated | NA | NA | NA | NA | Death | Seizure, coma |
| 71 | Exon 6 | c.594C>A | p.Asn198Lys | PAT | Zhou Duo(2023) | neonate | Neonatal | M | 410 | 2.44 | NA | NA | Elevated | NA | 3.2 | NA | 57 | 78 | NA | NA | NA | Alive | Poor feeding, growth and development lag behind |
| 72 | Exon 6 | c.595A>G | p.Asn199Asp | PAT | Liu Fang(2021) | 1D | Neonatal | M | 180 | 4.5 | 110.88 | 18.8 | NA | NA | NA | NA | NA | NA | NA | NA | NA | Death | pulmonary bleeding, upper gastrointestinal bleeding, convulsions and coma |
| 73 | Exon 6 | c.604C>T | p.His202Tyr | PAT | Deyun Lu (2020) | 4M | Late | M | 350 | 6.83 | 44.39 | 228.79 | 311.67 | NA | NA | NA | 28 | NA | NA | NA | NA | Alive | NA |
|  |  |  |  |  | Zhou Duo(2023) | neonate | Neonatal | F | 165 | 3.95 | NA | Elevated | Elevated | NA | 2.1 | NA | 297 | 149 | NA | NA | NA | Alive | Liver function impairment, convulsions, and transient blindness |
| 74 | Exon 6 | c.604dupC | p.L201fs | PAT | Liu Yanping(2019) | 2Y3M | Late | F | 532 | Reduce | Elevated | Elevated | Elevated | Normal | Normal | Normal | 112 | 66 | Normal | G3P2 | NA | Death | Titching, coma, tolerance, vomiting |
| 75 | Exon 6 | c.607T>A | p.Ser203Tyr | PAT | Xie Lei(2023) | 10Y4M | Late | M | 983.6 | Normal | Normal | Elevated | Elevated | NA | NA | NA | NA | 127 | NA | G1P1 | NA | Alive | Disorder of consciousness, convulsions, and vomiting |
| 76 | Exon 6 | c.612_614del | p.Ile204del | PAT | Yan(2024) | 1Y6M | Late | F | 360 | NA | NA | Elevated | Elevated | 7.6 | 5.4 | 0.6 | 883 | 1561 | 20.55 | G4P2 | 39 | Alive | Repeated vomiting was associated with restlessness |
| 77 | Exon 6 | c.613A>G | p.Met205Val | Likely PAT | Zhao Jing(2019)  Wang Ming(2023) | 3Y6M | Late | F | 214.8 | 17.59 | NA | 2.54 | 14.74 | NA | NA | NA | 150.5 | 103.9 | NA | NA | NA | Alive | Sleepiness, vomiting |
| 78 | Exon 6 | c.622G>A | p.Ala208Thr | PAT | Deyun Lu (2020) | 36Y | Late | M | 427 | 3.87 | 17.93 | ND | ND | NA | NA | NA | 36 | NA | NA | NA | NA | Alive | NA |
|  |  |  |  |  | Wu Feifei(2018) | 13Y5M | Late | M | 245.9 | 12.07 | 31.73 | 85 | 28.1 | NA | 1.38 | NA | 162 | 103 | 90.1 | NA | NA | Alive | Vomiting, and a disturbance of consciousness |
|  |  |  |  |  | Li Ruomeng(2024) | 15Y | Late | M | 250.6 | NA | NA | NA | NA | NA | NA | NA | 65 | 44 | 28.4 | NA | NA | Alive | Vomiting, disturbance of consciousness, and coma |
| 79 | Exon 6 | c.626C>T | p.Ala209Val | PAT | Min-Zhi Peng(2020) | 1Y6M | Late | F | NA | NA | NA | NA | NA | NA | NA | NA | NA | NA | NA | NA | NA | Alive | Recurrent vomiting, irritability, psychomotor retardation, talipes valgus |
|  |  |  |  |  | Deyun Lu (2020) | 1.5Y | Late | F | 133 | 15.9 | 23.87 | 457.51 | 112.32 | NA | NA | NA | ND | NA | NA | NA | NA | Alive | NA |
|  |  |  |  |  | Deyun Lu (2020) | 2.3Y | Late | F | 300 | 15.66 | 37.02 | 251.06 | 42.7 | NA | NA | NA | ND | NA | NA | NA | NA | Alive | NA |
|  |  |  |  |  | Qingnv Zhou(2020) | 2D | Neonatal | M | 704.3 | 3.15 | 156.6 | 80.5 | NA | 7.45 | 7.3 | -11.4 | 23 | NA | NA | NA | 36^+1^ | NA | Seizures,Somnolence,Acute encephalopathy, Coma, Fever, Feeding difficulties,Hypothermia,Cyanosis |
|  |  |  |  |  | Qingnv Zhou(2020) | 3D | Neonatal | M | 228.6 | 3.04 | 100.62 | 12.5 | 16.3 | 7.45 | 7 | -5.9 | 5 | NA | NA | NA | 36^+1^ | NA | Seizures,Somnolence,Acute encephalopathy,Coma,Fever, Feeding difficulties,Hypothermia |
|  |  |  |  |  | Liu Ning(2020) | ＜3M | Late | M | NA | NA | NA | NA | NA | NA | NA | NA | NA | NA | NA | NA | NA | Death | NA |
| 80 | Exon 6 | c.635G>T | p.Gly212Val | Likely PAT | Wang Jingwen(2023) | 2Y11M | Late | M | 280 | 5.43 | NA | 342.6 | 184.77 | NA | 4.5 | NA | 874 | 247 | NA | NA | NA | Alive | Fever, convulsions, and disorders of consciousness |
| 81 | Exon 6 | c.633+2T>G |  | likely pathogenic | Yan Beibei(2021) | 3D | Neonatal | M | 725 | 5.21 | NA | NA | NA | 7.49 | 6.6 | -5.8 | 223 | 2049 | NA | NA | 38^+3^ | Death | Fever, convulsions, and coma |
| 82 | Exon 6 | c.641A>C | p.His214Pro | PAT | Zhou Keke(2021) | 18Y | Late | F | 317 | NA | NA | 14.33 | 22.81 | NA | NA | NA | Normal | Normal | 34.6 | NA | NA | Alive | Episdic mental behavior abnormalities |
| 83 | Exon 6 | c.641A>G | p.His214R | PAT | Lan Liuting(2023) | 1Y5M | Late | F | 233.5 | NA | NA | NA | NA | 7.36 | 3.15 | -2.2 | 622 | 374 | NA | NA | NA | Alive | Vomiting, convulsions, and disorders of consciousness |
| 84 | Exon 6 | c.663G>A | p.Lys221= | PAT | Min-Zhi Peng(2020) | 3Y | Late | M | NA | NA | NA | NA | NA | NA | NA | NA | NA | NA | NA | NA | NA | Alive | Abdominal pain, vomiting, dysphoria, generalized seizure |
|  |  |  |  |  | 85Min-Zhi Peng(2020) | 5Y7M | Late | M | NA | NA | NA | NA | NA | NA | NA | NA | NA | NA | NA | NA | NA | Alive | Recurrent vomiting, middle coma |
|  |  |  |  |  | M86in-Zhi Peng(2020) | 1Y2M | Late | M | NA | NA | NA | NA | NA | NA | NA | NA | NA | NA | NA | NA | NA | Alive | Recurrent vomiting, lethargy, dysphoria |
| 85 | Intron 6 | c.664-1G>A | - | PAT | Deyun Lu (2020) | 6Y | Late | F | 170 | 10.4 | 42.54 | 199.3 | ND | NA | NA | NA | 338.2 | NA | NA | NA | NA | Alive | NA |
| 86 | Intron 6 | c.664-2(IVS6)A>C | - | PAT | Wang Jingwen(2023) | 1Y5M | Late | F | 217 | 10.85 | NA | 0 | 0 | NA | 4.5 | NA | 535 | 279 | NA | NA | NA | Alive | Vomiting, and abnormal liver function |
| 87 | Exon 7 | c.674C>T | p.Pro225Leu | PAT | Yongxian Shao(2017) | 1D | Neonatal | M | 1877 | 4.8 | 1802 | NA | NA | NA | NA | NA | Elevated | NA | NA | NA | NA | Death | Cyanosis |
|  |  |  |  |  | Yongxian Shao(2017) | 4D | Neonatal | M | 1048 | 3.4 | 3449 | NA | NA | NA | NA | NA | Normal | NA | NA | NA | NA | Death | Reduced intake |
|  |  |  |  |  | Min-Zhi Peng(2020) | 2D | Neonatal | M | NA | NA | NA | NA | NA | NA | NA | NA | NA | NA | NA | NA | NA | Death | Coma |
|  |  |  |  |  | Min-Zhi Peng(2020) | 3D | Neonatal | M | NA | NA | NA | NA | NA | NA | NA | NA | NA | NA | NA | NA | NA | Death | Generalized seizure, light coma |
|  |  |  |  |  | Liu Ning(2020) | ＜3M | Neonatal | F | NA | NA | NA | NA | NA | NA | NA | NA | NA | NA | NA | NA | NA | Death | NA |
| 88 | Exon 7 | c.703C>T | p.Gln235* | NR | Deyun Lu (2020) | 1.5Y | Late | F | 350 | 1.77 | ND | ND | ND | NA | NA | NA | 1455 | NA | NA | NA | NA | Alive | NA |
| 89 | Exon 7 | c.704A>C | p.Gln235Pro | NR | Deyun Lu (2020) | 3Y | Late | F | 286 | 10.91 | 37.1 | 356.42 | 42.7 | NA | NA | NA | 422 | NA | NA | NA | NA | Alive | NA |
| 90 | Intron 7 | c.717+1G>A | - | PAT | Hua Xia(2016)  Li Jun(2017)  Zhao Jing(2019) | 2Y6M | Late | F | 413 | 10.868 | NA | 374.83 | 245.22 | NA | 4.9 | NA | 174.3 | 177.4 | NA | NA | NA | Alive | Coma, reduced speech, convulsions, and vomiting |
| 91 | Intron 7 | c.717+2T>C | - | PAT | Zhu Yanfeng(2014) | 5Y | Late | F | 338 | NA | NA | Elevated | NA | NA | NA | NA | 434 | 203 | NA | NA | NA | NA | Repeated vomiting and abnormal liver function |
| 92 | Intron 7 | c． 717 + 6 _7het  _indelTAATAATAG | - | PAT | Zhu Yanfeng(2014) | 5Y | Late | F | 338 | NA | NA | Elevated | NA | NA | NA | NA | 434 | 203 | NA | NA | NA | NA | Repeated vomiting and abnormal liver function |
| 93 | Intron 7 | c.718-2A>G | - | PAT | Min-Zhi Peng(2020) | 1Y | Late | F | NA | NA | NA | NA | NA | NA | NA | NA | NA | NA | NA | NA | NA | Alive | Recurrent vomiting accompanied with lethargy, irritability |
| 94 | Intron 7 | c.718-1G>A | - | NR | Deyun Lu (2020) | 2Y | Late | F | 278 | 15.56 | 17.72 | 305.73 | 48.85 | NA | NA | NA | 964 | NA | NA | NA | NA | Alive | NA |
| 95 | Exon 8 | c.725C>T | p.Thr242Ile | PAT | Huang Junzi(2024) | Neonate | Neonatal | M | 925 | 6.23 | NA | 17.46 | NA | NA | NA | NA | NA | NA | NA | NA | 38^+1^ | Death | NA |
|  |  |  |  |  | Deyun Lu (2020) | 9D | Neonatal | M | 815 | 4.05 | ND | ND | ND | NA | NA | NA | ND | NA | NA | NA | NA | Death | NA |
| 96 | Exon 8 | c.755G>T | p.Val252Leu | NR | Min-Zhi Peng(2020) | 1Y7M | Late | M | NA | NA | NA | NA | NA | NA | NA | NA | NA | NA | NA | NA | NA | Death | Recurrent vomiting, complex partial seizures, middle coma |
| 97 | Exon 8 | c.761C>A | p.A254E | VUS | Yan Beibei(2021) | 3D | Neonatal | M | 2698 | 2.93 | NA | 315.5 | NA | 7.51 | 10.2 | -4.6 | 32 | 98 | NA | G3P2 | 39^+2^ | Death | Poor response, convulsions, and coma |
| 98 | Exon 8 | c.779T>C | p.Leu260Ser | PAT | Deyun Lu (2020) | 1.1Y | Late | F | 260 | 4.27 | ND | 469.38 | 261.67 | NA | NA | NA | 72 | NA | NA | NA | NA | Death | NA |
|  |  |  |  |  | Zhou Duo(2023) | 7D | Neonatal | M | NA | 4.94 | NA | NA | NA | NA | NA | NA | NA | NA | NA | NA | NA | Death | NA |
| 99 | Exon 8 | c.782T>C | p.Ile261Thr | NR | Li Sitao(2018) | 7D | Neonatal | M | 700 | 1.77 | NA | 33.11 | 2.15 | NA | NA | NA | NA | NA | NA | NA | NA | Death | Poor mental reactions, vomiting, convulsions |
| 100 | Exon 8 | c.784_792dup | p.Thr262_Thr264dup | NR | Yi Lijun(2018) | 3D | Neonatal | M | 1539 | Reduce | NA | Elevated | Elevated | 7.1 | NA | 4 | NA | NA | NA | G1P1 | 40 | Death | Poor response, poor error, twitching |
| 101 | Exon 8 | c.785C>T | p.Thr262Ile | PAT | Yongxian Shao(2017) | 9M | Late | M | 349 | 4.3 | 858 | NA | NA | NA | NA | NA | Elevated | NA | NA | NA | NA | Alive | Vomiting,growth retardation |
|  |  |  |  |  | Min-Zhi Peng(2020) | 6M | Late | M | NA | NA | NA | NA | NA | NA | NA | NA | NA | NA | NA | NA | NA | Alive | Recurrent vomiting, psychomotor regression |
|  |  |  |  |  | Mo Weiqian(2011) | 6M | Late | M | 203.5 | 9 | 753 | Elevated | Elevated | 7.505 | NA | NA | 466 | 355 | NA | G1P1 | NA | NA | Sitting alone is unstable, vomiting |
| 102 | Exon 8 | c.793T>G | p.W265G | PAT | Wang Ming(2023) | 1Y3M | Late | F | 255.8 | 11.09 | NA | 354.98 | 5.78 | NA | NA | NA | 168.3 | NA | NA | NA | NA | Alive | NA |
| 103 | Exon 8 | c.794G>A | p.Trp265* | NR | Deyun Lu (2020) | 4.3Y | Late | M | 114 | 21.74 | 16.37 | 312.35 | 248.4 | NA | NA | NA | 244 | NA | NA | NA | NA | Death | NA |
| 104 | Exon 8 | c.800G>C | p.Ser267Tyr | PAT | Chen Zhanling(2014) | 1Y | Late | M | 1120 | 2.5 | NA | Elevated | Elevated | NA | 3.14 | NA | NA | NA | NA | NA | NA | Death | Fever, respiratory failure, and brain herniation |
|  |  |  |  |  | Liu Li(2022) | Neonate | Neonatal | NA | NA | 4.04 | NA | NA | NA | NA | NA | NA | NA | NA | NA | NA | NA | NA | Newborn screening has confirmed the diagnosis |
| 105 | Exon 8 | c.803T>C | p.Met268Thr | PAT | Qingnv Zhou(2020) | 6D | Neonatal | M | 1030 | 2.71 | 1577.35 | 352.13 | 32.03 | 7.43 | 3 | -1.3 | 25 | NA | NA | NA | 38^+1^ | NA | Seizures,Somnolence,Acute encephalopathy, Coma, Fever, Feeding difficulties |
|  |  |  |  |  | Wang Haijun(2018) | 10M | Late | M | 343.5 | 4.77 | NA | 489.61 | 122.043 | 7.413 | NA | NA | 271 | 68 | NA | NA | NA | Alive | Vomiting, convulsions, disorders of consciousness |
|  |  |  |  |  | Chu Xiaoyun(2023) | 8D | Neonatal | M | 700 | 2.7 | 1577.3 | 352.1 | 32.0 | NA | 3.0 | NA | NA | NA | NA | NA | 38^+1^ | Alive | Vomiting, drowsiness |
| 106 | Exon 8 | c.805G>A | p.Gly269Arg | NR | Yongxian Shao(2017) | 11M | Late | M | 186 | 7.2 | 722 | NA | NA | NA | NA | NA | Elevated | NA | NA | NA | NA | Alive | Vomiting,growth retardation |
|  |  |  |  |  | Zhan Yishan(2020) | 7M | Late | M | 500 | Reduce | NA | Elevated | NA | NA | NA | NA | 140 | 57 | NA | G5P5 | NA | Death | Vomiting, convulsions, and disorders of consciousness |
|  |  |  |  |  | Min-Zhi Peng(2020) | 9D | Neonatal | M | NA | NA | NA | NA | NA | NA | NA | NA | NA | NA | NA | NA | NA | Alive | Generalized seizure |
| 107 | Exon 8 | c.829C>T | p.Arg277Trp | PAT | Yongxian Shao(2017) | 6Y | Late | M | 202 | 15 | 838 | NA | NA | NA | NA | NA | Elevated | NA | NA | NA | NA | Alive | Irritability |
|  |  |  |  |  | Wenjia Tong(2018) | 4Y | Late | M | NA | NA | NA | NA | NA | NA | NA | NA | NA | NA | NA | NA | NA | NA | Fever, Vomiting, Coma, Generalized tonic-clonic seizures, Muscular hypotonia, Dyspnea, Cerebral edema, Trombocytosis |
|  |  |  |  |  | Deyun Lu (2020) | 1.4Y | Late | M | 351 | 5.62 | 80.6 | 413.21 | 143.06 | NA | NA | NA | 18 | NA | NA | NA | NA | Death | NA |
|  |  |  |  |  | Deyun Lu (2020) | 2.6Y | Late | M | 244 | ND | 9.92 | ND | ND | NA | NA | NA | 55 | NA | NA | NA | NA | Alive | NA |
|  |  |  |  |  | Deyun Lu (2020) | 1.4Y | Late | M | 114 | 25.43 | ND | 137 | 6.07 | NA | NA | NA | ND | NA | NA | NA | NA | Alive | NA |
|  |  |  |  |  | Deyun Lu (2020) | 9.7Y | Late | M | 120 | 8.58 | 5.61 | 96 | 42.9 | NA | NA | NA | 40 | NA | NA | NA | NA | Alive | NA |
|  |  |  |  |  | Deyun Lu (2020) | 10M | Late | M | 131 | 2.28 | 11.23 | ND | ND | NA | NA | NA | 69 | NA | NA | NA | NA | Alive | NA |
|  |  |  |  |  | Deyun Lu (2020) | 3Y | Late | M | 289 | 7.48 | ND | 48.31 | 165.31 | NA | NA | NA | 327 | NA | NA | NA | NA | Death | NA |
|  |  |  |  |  | Deyun Lu (2020) | 3D | Neonatal | M | 700 | 2.48 | 22.23 | 267.6 | 53.26 | NA | NA | NA | 119 | NA | NA | NA | NA | Alive | NA |
|  |  |  |  |  | Min-Zhi Peng(2020) | 12Y | Late | M | NA | NA | NA | NA | NA | NA | NA | NA | NA | NA | NA | NA | NA | Death | Vomiting, middle coma, Generalized seizure |
|  |  |  |  |  | Min-Zhi Peng(2020) | 2Y | Late | M | NA | NA | NA | NA | NA | NA | NA | NA | NA | NA | NA | NA | NA | Alive | Irritability, recurrent vomiting accompanied with lethargy, abdominal pain |
|  |  |  |  |  | Min-Zhi Peng(2020) | 4Y | Late | M | NA | NA | NA | NA | NA | NA | NA | NA | NA | NA | NA | NA | NA | Alive | Vomiting accompanied with lethargy, middle coma |
|  |  |  |  |  | Zhao Jing(2019),Wang Ming(2023) | 5Y6M | Late | M | 528 | NA | NA | NA | NA | NA | NA | NA | 104.6 | 200.1 | NA | NA | NA | Death | Coma, convulsions, vomiting, biting, nonsense |
|  |  |  |  |  | Wang Ming(2023) | 3Y2M | Late | M | 243.9 | 5.79 | NA | 189.21 | 72.36 | NA | NA | NA | 81 | NA | NA | NA | NA | Alive | NA |
|  |  |  |  |  | Wang Ming(2023) | 1Y6M | Late | M | 779.6 | 6.24 | NA | 1140.9 | 75.93 | NA | NA | NA | 40 | NA | NA | NA | NA | Death | NA |
|  |  |  |  |  | Zhao Jing(2019) | 2Y7M | Late | M | 243 | 5.79 | NA | 189.21 | 72.36 | NA | NA | NA | 180.2 | 148.4 | NA | NA | NA | Alive | Coma, irritable, cry and scream |
|  |  |  |  |  | Mo Weiqian(2011) | 2Y | Late | M | 202.9 | 15 | 838 | Elevated | Elevated | Normal | Normal | Normal | 44 | NA | NA | G1P1 | NA | NA | Irritable, vomiting |
|  |  |  |  |  | Nan Zaiyuan(2020) | 2Y | Late | M | Normal | NA | NA | 481.69 | 510.94 | NA | Normal | NA | Normal | Normal | Normal | G2P2 | NA | Alive | Vomiting, drowsiness, fever, and twitch |
|  |  |  |  |  | Wang Jingwen(2023) | 1Y8M | Late | M | 172.1 | 5.49 | NA | 0 | 120.87 | NA | 5.2 | NA | 67 | 45 | NA | NA | NA | Alive | Vomiting, drowsiness, and twitching |
| 108 | Exon 8 | c.830G>A | p.Arg277Gln | PAT | Mak(2007) | 3Y | Late | M | 54 | NA | 747 | 700 | NA | NA | NA | NA | NA | NA | NA | NA | NA | Alive | Acute encephalopathy, acoma |
|  |  |  |  |  | Wang Ming(2023) | 7Y1M | Late | M | 196.2 | 11.24 | NA | NA | 0.52 | NA | NA | NA | 63.3 | NA | NA | NA | NA | Alive | NA |
| 109 | Exon 8 | c.852C>G | p.Tyr284* | NR | Wu Bo (2018) | 14M | Late | F | 94 | Normal | NA | Elevated | Elevated | 7.4 | 6.22 | -3.1 | 117 | 71 | NA | NA | NA | Alive | Sleepy and vomiting with limb tremor |
| 110 | Exon 8 | c.860C>T | p.Thr287Ile | NR | Deyun Lu (2020) | 5D | Neonatal | M | 50 | 3.24 | 15.15 | 22.28 | 8.7 | NA | NA | NA | 172 | NA | NA | NA | NA | Alive | NA |
| 111 | Exon 8 | c.865A>T | p.K289X | NR | Wang Ming(2023) | 2Y9M | Late | F | 547 | 10.89 | 4.348 | 348.69 | 185.9 | NA | 3 | NA | 144.7 | 65.9 | NA | NA | NA | Alive | Ingety, abnormal behavior, limb tremor, drowsiness, urinary incontinence |
|  |  |  |  |  | Hua Xia(2016)  Li Jun(2017)  Zhao Jing(2019) | 2Y3M | Late | F | 459 | 8.519 | NA | 250.12 | 133.27 | NA | NA | NA | 788.4 | 637.8 | NA | NA | NA | Alive | Sleepgy, sleep disturbance, behavioral abnormalities, vomiting, mental regression |
| 112 | Intron 8 | c.867+1G>C | - | NR | Li Sitao(2018) | NA | Neonatal | M | 600 | 3.66 | NA | 41.6 | NA | NA | NA | NA | NA | NA | NA | NA | NA | Death | Poor feeding, poor mental distress, coma, and drowsiness |
| 113 | Intron 8 | c.868-1G>C | - | NR | Deyun Lu (2020) | 4Y | Late | F | >500 | 5.69 | 23.48 | 19.93 | 338.64 | NA | NA | NA | 839 | NA | NA | NA | NA | Alive | NA |
| 114 | Exon 9 | c.893G>C | p.Trp298Ser | PAT | Zhao Jing(2019) | 8Y6M | Late | F | 175.6 | 13.27 | NA | 433.63 | 88.01 | NA | NA | NA | 41.8 | 37.5 | NA | NA | NA | Alive | Disorder of consciousness, vomiting, abnormal behavior, |
|  |  |  |  |  | Wang Ming(2023) | 8Y6M | Late | F | 175.6 | 13.27 | NA | 1445 | 31 | NA | NA | NA | 41.8 | NA | NA | NA | NA | Alive | NA |
| 115 | Exon 9 | c.893G>A | p.Trp298* | NR | Min-Zhi Peng(2020) | 2Y10M | Late | F | NA | NA | NA | NA | NA | NA | NA | NA | NA | NA | NA | NA | NA | Alive | Recurrent vomiting |
| 116 | Exon 9 | c.898delT | - | PAT | Wang Haijun(2018) | 5Y5M | Late | M | 854 | 12.343 | NA | 1356.425 | 144.467 | 7.443 | NA | NA | 255 | 168 | NA | NA | NA | Death | Vomiting, disturbance of consciousness, ataxia |
|  |  |  |  |  | Liu Ning（2020） | 5Y | Late | F | NA | NA | NA | NA | NA | NA | NA | NA | NA | NA | NA | NA | NA | Alive | NA |
| 117 | Exon 9 | c.898dupT | - | PAT | Wang Haijun(2018) | 3Y7M | Late | F | 353 | 6.188 | NA | 8730.249 | 94.906 | 7.383 | NA | NA | 32 | 19 | NA | NA | NA | Death | Fever, poor tolerance, vomiting, delayed psychomotor development, and muscular hypotonia |
| 118 | Exon 9 | c.904C>T | p.His302Tyr | PAT | Deyun Lu (2020) | 1D | Neonatal | M | >500 | 2.18 | 24.79 | 127.33 | 90.78 | NA | NA | NA | 15 | NA | NA | NA | NA | Death | NA |
| 119 | Exon 9 | c.912G>T | p.Leu304Phe | PAT | Fang Weiyuan(2023) | 3Y8M | Late | F | 57 | 16.71 | NA | 0 | 0 | Normal | Normal | Normal | 339 | 87 | 6.1 | G1P1 | NA | Alive | Dysfunction of blood coagulation |
| 120 | Exon 9 | c.913C>T | p.Pro305Ser | NR | Deyun Lu (2020) | 7M | Late | M | 392 | 1.1 | 14.28 | 543.54 | 130.02 | NA | NA | NA | 462 | NA | NA | NA | NA | Alive | NA |
| 121 | Exon 9 | c.914C>G | p.Pro305Arg | PAT | Deyun Lu (2020) | 1.8Y | Late | F | 216 | 5.94 | NA | 204.05 | 83.3 | NA | NA | NA | 885 | NA | NA | NA | NA | Alive | NA |
| 122 | Exon 9 | c.917G>C | p.Arg306Thr | NR | Meng Lulu (2013) | 48H | Neonatal | M | 479 | 3.09 | 133.65 | 12.86 | NA | NA | NA | NA | NA | NA | NA | NA | NA | Death | Low reaction |
| 123 | Exon 9 | c.919A>G | p.Lys307Glu | VUS | Deyun Lu (2020) | 1Y | Late | M | 45 | 7.09 | 14.21 | 1.85 | 19.79 | NA | NA | NA | 26 | NA | NA | NA | NA | Alive | NA |
| 124 | Exon 9 | c.929_931del | p.Glu310Valfs*45 | NR | Deyun Lu (2020) | 2.1Y | Late | M | 257 | 12.77 | 13.53 | 11.6 | 17.53 | NA | NA | NA | 116 | NA | NA | NA | NA | Alive | NA |
|  |  |  |  |  | Wang Jingwen(2023) | 1Y7M | Late | F | 348 | 10.34 | NA | 183.83 | 1207.26 | NA | 6.2 | NA | 568 | 404 | NA | NA | NA | Alive | Vomiting |
| 125 | Exon 9 | c.929A>G | p.Glu310Gly | PAT | Min-Zhi Peng(2020) | 9M | Late | M | NA | NA | NA | NA | NA | NA | NA | NA | NA | NA | NA | NA | NA | Alive | Psychomotor retardation, recurrent vomiting, light coma, irritability |
|  |  |  |  |  | Yongxian Shao(2017) | 11M | Late | M | 100 | 5.6 | 770 | NA | NA | NA | NA | NA | Elevated | NA | NA | NA | NA | Alive | Vomiting, seizure |
| 126 | Exon 9 | c.931G>A | p.Val311Met | PAT | Deyun Lu (2020) | 1.2Y | Late | M | 187 | 3.86 | 15.48 | 455.63 | 133 | NA | NA | NA | 48 | NA | NA | NA | NA | Alive | NA |
|  |  |  |  |  | Zhou Duo(2023) | neonate | Asymptomatic | M | normal | 4.33 | NA | NA | NA | NA | Normal | NA | Normal | Normal | NA | NA | NA | Alive | Asymptomatic |
| 127 | Exon 9 | c.944T>G | p.Val315Gly | PAT | Deyun Lu (2020) | 10M | Late | F | 315 | ND | ND | ND | ND | NA | NA | NA | 885 | NA | NA | NA | NA | Alive | NA |
| 128 | Exon 9 | c.958C>T | p.Arg320* | PAT | Li Yunning（2023） | 3D | Neonatal | M | 478 | 3.3 | NA | 64.9 | NA | NA | NA | NA | NA | 115.63 | 128.62 | NA | 40 | Death | Sleepiness, coma |
|  |  |  |  |  | Zhang Qinghua(2021) | Neonatal | Neonatal | M | NA | NA | NA | NA | NA | NA | NA | NA | NA | NA | NA | NA | NA | NA | Neonatal convulsions, metabolic acidosis, and hyperaminaemia |
|  |  |  |  |  | Sun Weihua(2011) | 3D | Neonatal | M | 235 | 1.06 | NA | 124 | NA | 7.17 | 5.3 | NA | 52 | 32 | 312.4 | G1P1 | 41^+2^ | Death | Poor response, lethargy, dyspnea, and convulsions |
| 129 | Exon 9 | c.961T>C | p. Ser321Pro | NR | Aizhen Yu(2023) | 3D | Neonatal | M | 2518.4 | 4.31 | 137.59 | 203.5 | 7.1 | NA | 10.18 | NA | 122 | 32 | NA | G1P1 | 39^+6^ | Death | Decmuscle tone, difficulty breathing, and convulsions |
|  |  |  |  |  | Wu Feifei(2018) | 3D | Neonatal | M | 2518.4 | 4.31 | 137.59 | 203.5 | 7.1 | NA | 12.9 | NA | 1290 | 2779 | 510.8 | NA | NA | Death | Titching, and abnormal muscle tone |
| 130 | Exon 9 | c. 959G ＞ C | p．R320P | NA | Tang Jianping(2017) | 8D | Neonatal | M | NA | NA | NA | Elevated | Elevated | NA | Elevated | NA | NA | NA | NA | NA | NA | NA | High lactic acid, high blood ammonia, and tics |
| 131 | Exon 9 | c.970_979delTTCCCAGAGG | p.Phe324Glnfs*16 | Likely PAT | Wang Liping(2022) | 5D | Neonatal | M | 461 | NA | NA | NA | NA | NA | 10.8 | NA | NA | NA | NA | NA | 40 | Death | Stopped feeding, showed no movement, and exhibited poor responsiveness, which was accompanied by an abnormal increase in muscle tone, shortness of breath, moaning, foaming at the mouth, screaming, pumping, vomiting, abdominal distention, and blood in the stool. |
| 132 | Exon 9 | c.995G>C | p.Trp332Ser | NR | Wang Yan(2014) | 9D | Neonatal | M | 1000 | 2.67 | 161.05 | Elevated | Elevated | 7.42 | NA | -0.5 | NA | NA | NA | NA | NA | Death | Poor mental response and feeding difficulties |
| 133 | Exon 9 | c.1001C > A | p.Leu201Met | NR | Min-Zhi Peng(2020) | 1Y | Late | F | NA | NA | NA | NA | NA | NA | NA | NA | NA | NA | NA | NA | NA | Death | Complex partial seizures |
| 134 | Intron 9 | c.1005+1G>A | - | NR | Yongxian Shao(2017) | 2Y | Late | F | 364 | 13.5 | 1041 | NA | NA | NA | NA | NA | Elevated | NA | NA | NA | NA | Death | Vomiting |
|  |  |  |  |  | Min-Zhi Peng(2020) | 2Y5M | Late | F | NA | NA | NA | NA | NA | NA | NA | NA | NA | NA | NA | NA | NA | Death | Recurrent vomiting, irritability |
| 135 | Intron 9 | c.1005+132InsT | - | NR | Sun Weihua（2011） | 3D | Neonatal | M | 235 | 1.06 | NA | 124 | NA | 7.17 | 5.3 | NA | 52 | 32 | 312.4 | G1P1 | 41^+2^ | Death | Poor response, lethargy, dyspnea, and convulsions |
| 136 | Intron 9 | c.1005+3172 T>C | - | NR | Chen Zhehui(2022) | 5Y10M | Late | F | 108 | 10.05 | NA | 3.3 | 31.7 | NA | NA | NA | 127 | 91 | NA | G3P1 | NA | Alive | Vomiting, liver injury |
| 137 | Intron 9 | c.1005+1025A>G | - | NR | Liu Li(2022) | Neonate | Neonatal | NA | NA | 6.33 | NA | NA | NA | NA | NA | NA | NA | NA | NA | NA | NA | NA | Newborn screening has confirmed the diagnosis |
| 138 | Intron 9 | c.1006C>G | - | NR | Li Xuebo(2020) | 4D | Neonatal | M | Elevated | NA | Elevated | NA | NA | NA | Elevated | NA | NA | NA | NA | NA | NA | Death | Vomiting, difficulty breathing, drowsiness, and coma |
| 139 | Intron 9 | c.1006-3C>G | - | PAT | Xu Yuping(2019) | 8y | Late | M | 510 | 4.95 | NA | 92.18 | 152.99 | NA | 8.2 | NA | NA | NA | NA | NA | NA | Alive | Vomiting, abdominal pain, and poor response |
| 140 | Exon 10 | c.1015G>A | p.Val339M | NR | Zhang Yanghui(2014) | NA | NA | NA | NA | NA | NA | NA | NA | NA | NA | NA | NA | NA | NA | NA | NA | NA | NA |
| 141 | Exon 10 | c.1016T>G | p.Val339Gly | NR | Wang Yan (2014) | 7D | Neonatal | M | 1000 | 4.95 | 434.09 | 92.18 | 1382.94 | 7.17 | NA | -9.2 | NA | NA | NA | G4P3 | NA | Death | Poor response, and twitching |
| 142 | Exon 10 | c.1019C>T | p.Ser340Phe | VUS | Deyun Lu (2020) | 8M | Late | M | 335 | 13.6 | ND | 82.82 | 339.11 | NA | NA | NA | 1782 | NA | NA | NA | NA | Alive | NA |
|  |  |  |  |  | Zhou Duo(2023) | neonate | Late | M | 412 | 3.1 | NA | NA | Elevated | NA | 4.6 | NA | 1138 | 382 | NA | NA | NA | Alive | Vomiting, poor tolerance, drowsiness, and convulsions |
| 143 | Exon 10 | c.1028C>A | p.Thr343Lys | PAT | Zhao Jing(2019)  Wang Ming(2023) | 6M22D | Late | F | 262 | 1.66 | NA | 1123.5 | 239.8 | NA | NA | NA | 64.4 | 97.6 | NA | NA | NA | Alive | Coma, convulsions, involuntary movements, and vomiting |
| 144 | Exon 10 | c.1028C>G | p.Thr343Arg | VUS | Wang Jingwen(2023) | 8M | Late | F | 183 | 4.61 | NA | 0 | 0 | NA | 3.3 | NA | 1094 | 2801 | NA | NA | NA | Alive | Fever |
| 145 | Exon 10 | c.1048C>T | p.Q350X | VUS | Wang Ming(2023) | 4D | Neonatal | M | 818.6 | 2.791 | NA | 161.89 | NA | NA | NA | NA | 22 | NA | NA | NA | NA | Death | NA |
| 146 | Exon1-4 | del | - | PAT | Gong Zhuwen(2016) | 2Y | Late | F | 383.5 | 3.7 | NA | 55.5 | NA | NA | NA | NA | NA | NA | NA | NA | NA | NA | Recurrent vomiting |
|  |  |  |  |  | Deyun Lu (2020) | 1Y | Late | F | 383 | 3.7 | 5.18 | 55.46 | 156.57 | NA | NA | NA | 49 | NA | NA | NA | NA | Alive | NA |
| 147 | Exon1-4 | dup | - | PAT | Gong Zhuwen(2016) | 6Y | Late | M | 2500.1 | 2.8 | NA | 172.8 | NA | NA | NA | NA | NA | NA | NA | NA | NA | Death | Recurrent vomiting |
|  |  |  |  |  | Deyun Lu (2020) | 5Y | Late | M | 2500 | 2.8 | ND | 172.8 | 27.8 | NA | NA | NA | 19.7 | NA | NA | NA | NA | Death | NA |
| 148 | Exon2-4 | del | - | PAT | Gong Zhuwen(2016) | 9Y | Late | F | 289.4 | 7.4 | NA | 106.7 | NA | NA | NA | NA | NA | NA | NA | NA | NA | NA | Recurrent vomiting |
|  |  |  |  |  | Deyun Lu (2020) | 9.3Y | Late | F | 268 | 7.44 | 56.51 | 106.88 | 61.87 | NA | NA | NA | ND | NA | NA | NA | NA | Withdraw | NA |
| 149 | Exon2-6 | dup | - | PAT | Gong Zhuwen(2016) | 10D | Neonatal | M | 780.7 | 4.8 | NA | 1638 | NA | NA | NA | NA | NA | NA | NA | NA | NA | Death | Vomiting, drowsiness, and coma |
|  |  |  |  |  | Deyun Lu (2020) | 3D | Neonatal | M | 780 | 4.8 | 43.73 | 1638.32 | 61.91 | NA | NA | NA | NA | NA | NA | NA | NA | Death | NA |
| 150 | Exon5-8 | del | - | NR | Deyun Lu (2020) | 1.7Y | Late | F | ＞500 | 11.54 | 20.67 | 139.1 | 107.17 | NA | NA | NA | 623 | NA | NA | NA | NA | Alive | NA |
| 151 | Exon3-9 | del |  | NR | Yan Beibei(2021) | 4D | Neonatal | M | 1546 | 2.82 | NA | 57.4 | NA | 7.29 | 11..9 | -11.2 | 13 | 124 | NA | G1P1 | 37^+2^ | Death | Stid of breath, edema, convulsions, and coma |
| 152 | Exon5 | 500bpSINE | - | Likely PAT | Aizhen Yu(2023) | 3D | Neonatal | M | 1550.1 | 1.96 | NA | 5.2 | NA | NA | NA | NA | NA | NA | NA | G7P5 | 38^+2^ | Death | Hypotonia, poor response, coma, and difficulty in breathing |
| 153 | Exon7-9 | del | - | PAT | Zhang Qinghua(2024) | 3D | Neonatal | F | 2244.9 | 4.09 | NA | 36.11 | NA | 7.292 | NA | -10.4 | NA | NA | NA | NA | NA | NA | Blue skin, no food |
| 154 | Exon7-10 | del | - | PAT | Gong Zhuwen(2016) | 20D | Neonatal | M | 1015.2 | 3.6 | NA | 158.3 | NA | NA | NA | NA | NA | NA | NA | NA | NA | Death | Feeding difficulties, vomiting, and drowsiness |
|  |  |  |  |  | Deyun Lu (2020) | 3D | Neonatal | M | 1015 | 4.96 | NA | 214.77 | 9.89 | NA | NA | NA | NA | NA | NA | NA | NA | Death | NA |
| 155 | Exon9-10 | del | p.Thr290_Phe354del | NR | Deyun Lu (2020) | 27D | Neonatal | F | 410 | 14.83 | 29.73 | 197 | 51.8 | NA | NA | NA | 2602 | NA | NA | NA | NA | Alive | NA |
| 156 | Exon1-10 | del | - | NR | Cui Qingyang(2021) | 3D | Neonatal | F | 1810 | 4.12 | NA | 166.3 | NA | 7.05 | 8.5 | -11 | 409 | 1100 | NA | G4P2 | NA | Death | Poor response, convulsions, unconscious disorders |
|  |  |  |  |  | Min-Zhi Peng(2020) | 1Y4M | Late | F | NA | NA | NA | NA | NA | NA | NA | NA | NA | NA | NA | NA | NA | Death | Recurrent vomiting accompanied with lethargy, avoidance of meat and milk, psychomotor retardation, hypotonia |
| 157 | Exon1-10 | c.214_*368{0}  delOTC gene >1.6 Mb | p.0 | PAT | Yongxian Shao(2017) | 3D | Neonatal | F | 4215 | 3.9 | 3282 | NA | NA | NA | NA | NA | Normal | NA | NA | NA | NA | Death | Seizure |
|  |  |  |  |  | Yongxian Shao(2017) | 1Y4M | Late | F | 665 | 15.3 | 1063 | NA | NA | NA | NA | NA | Elevated | NA | NA | NA | NA | Death | Vomiting, irritability |
|  |  |  |  |  | Min-Zhi Peng(2020) | 1D | Neonatal | M | NA | NA | NA | NA | NA | NA | NA | NA | NA | NA | NA | NA | NA | Death | Irritability, generalized seizure, severe coma |
| 158 | - | Xp11.4 7.8Mb del | - | NR | Deyun Lu (2020) | 3.4Y | Late | M | 447 | 18.62 | 14.04 | 77.3 | 62.9 | NA | NA | NA | 228.9 | NA | NA | NA | NA | Alive | NA |
| 159 | - | Xp21.1p11.4c×0 | Del | PAT | Wang Ming(2023) | 3D | Neonatal | M | 1893 | 2.629 | NA | 1316 | 19.75 | NA | NA | NA | 24 | NA | NA | NA | NA | Death | NA |

PAT: pathogenic; M: male; F: fenale; NA: Not Available; ND, not detected
