# Supplementary material for: Clinical and biochemical characteristics of patients with ornithine transcarbamylase deficiency and in silico analysis of OTC gene
Source: Orphanet J Rare Dis. 2025 Mar 18;20:131. doi: 10.1186/s13023-025-03624-4 (PMC11916849; doi:10.1186/s13023-025-03624-4)
Supplement: Supplementary file 2 — Supplementary Material 2 [file 13023_2025_3624_MOESM2_ESM.docx]

**S2-1** Top 10 OTC gene mutation sites in China in references

| Variant | Amino acid changes | Number(%) |
| --- | --- | --- |
| c.829C>T | p.Arg277Trp | 19 (6.53) |
| c.119G>A | p.Arg40His | 18 (6.19) |
| c.386G>A | p.Arg129His | 9 (3.09) |
| c.482A>G | p.Asn161Ser | 7 (2.41) |
| c.586G>A | p.Asp196Asn | 6 (2.06) |
| c.626C>T | p.Ala209Val | 6 (2.06) |
| c.583G>A | p.Gly195Arg | 6 (2.06) |
| c.674C>T | p.Pro225Leu | 5 (1.72) |
| c.422G>A | p.Arg141Gln | 5(1.72) |
| c.116G>T | p.Gly39Val | 4 (1.50) |
| c.78-2A>G | - | 4 (1.50) |

**S2-2** The phenotype distribution of Chinese OTCD patients in references

| Phenotype | | LO | EO | AS | Total |
| --- | --- | --- | --- | --- | --- |
| Females | | 101 | 8 | 0 | 109 |
| Males | | 99 | 76 | 2 | 177 |
| No mentioned | | 1 | 3 | 1 | 5 |
| Total | | 201 | 87 | 3 | 291 |
|  |  |  | LO | EO | ***P*** |
| Index | Ammonia  (μmol/L) | Number | 163 | 67 | ***＜0.0001*** |
|  |  | Median | 314.3 | 845.6 |  |
|  | Citrulline  (μmol/L) | Number | 135 | 67 | ***＜0.0001*** |
|  |  | Median | 8.820 | 3.600 |  |
|  | Glutamine  (μmol/L) | Number | 72 | 31 | **0.0053** |
|  |  | Median | 29.47 | 133.7 |  |
|  | Orotic acid  (mmol/molCr) | Number | 100 | 47 | 0.9662 |
|  |  | Median | 147.4 | 127.3 |  |
|  | Uracil  (mmol/molCr) | Number | 96 | 23 | ***0.0078*** |
|  |  | Median | 65.20 | 32.20 |  |
|  | PH | Number | 18 | 26 | 0.1448 |
|  |  | Median | 7.407 | 7.265 |  |
|  | Lac  (mmol/L) | Number | 32 | 30 | ***0.0001*** |
|  |  | Median | 4.235 | 7.350 |  |
|  | BE | Number | 6 | 23 | 0.8063 |
|  |  | Median | -4.2 | -5.0 |  |
|  | ALT  (U/L) | Number | 119 | 29 | ***＜0.0001*** |
|  |  | Median | 215.0 | 42.0 |  |
|  | AST  (U/L) | Number | 61 | 15 | 0.2922 |
|  |  | Median | 177.4 | 115.6 |  |
|  | TBIL  (μmol/L) | Number | 13 | 8 | ***＜0.0001*** |
|  |  | Median | 22.7 | 312.4 |  |

EO, early-onset; LO, late-onset; AS, Asymptomatic; Lac, lactic acid；ALT, alanine aminotransferase ; AST, glutamic-oxalacetic transaminase, TBIL, total bilirubin.

**S2-3** The phenotype and gender distribution of Chinese OTCD patients in references

|  |  | LO-F | LO-M | EO-F | EO-M | LO-F/M  ***P*** | EO-F/M  ***P*** | LO/EO-F  ***P*** | LO/EO-M  ***P*** |
| --- | --- | --- | --- | --- | --- | --- | --- | --- | --- |
| Ammonia  (μmol/L) | Median | 338.0 | 261.0 | 1810 | 832.1 | 0.6084 | 0.4529 | ***0.0139*** | ***＜0.0001*** |
|  | Number | 81 | 82 | 7 | 60 |  |  |  |  |
| Citrulline#  (μmol/L) | Median | 10.89 | 6.03 | 402 | 3.27 | ***0.0001*** | 0.3114 | ***0.0093*** | ***＜0.0001*** |
|  | Number | 69 | 66 | 6 | 58 |  |  |  |  |
| Glutamine  (μmol/L) | Median | 23.48 | 44.88 | 29.73 | 135.6 | 0.2851 | 0.7239 | 0.3706 | 0.1227 |
|  | Number | 37 | 35 | 3 | 28 |  |  |  |  |
| Orotic acid  (mmol/molCr) | Median | 146.2 | 158.3 | 181.7 | 124.0 | 0.5346 | 0.7245 | 0.9876 | 0.6698 |
|  | Number | 58 | 42 | 4 | 43 |  |  |  |  |
| Uracil  (mmol/molCr) | Median | 77.58 | 43.92 | 43.18 | 19.75 | 0.3742 | 0.5020 | 0.5968 | ***0.0223*** |
|  | Number | 54 | 42 | 2 | 21 |  |  |  |  |
| PH | Median | 7.387 | 7.436 | 7.292 | 7.240 | 0.5148 | 0.9908 | 0.3758 | 0.2479 |
|  | Number | 8 | 10 | 3 | 23 |  |  |  |  |
| Lac  (mmol/L) | Median | 3.3 | 4.55 | 7.4 | 7.3 | 0.1859 | 0.6392 | 0.4268 | ***0.0030*** |
|  | Number | 13 | 18 | 3 | 27 |  |  |  |  |
| BE | Median | -2.2 | -7.6 | -10.4 | -4.9 | 0.1000 | 0.3981 | 0.4000 | 0.4506 |
|  | Number | 3 | 3 | 3 | 20 |  |  |  |  |
| ALT  (U/L) | Median | 349 | 116 | 349 | 409 | ***0.0005*** | ***0.0060*** | 0.4022 | ***0.0003*** |
|  | Number | 65 | 53 | 65 | 3 |  |  |  |  |
| AST  (U/L) | Median | 203 | 160 | 624.5 | 98 | 0.5000 | 0.2857 | 0.5302 | 0.2039 |
|  | Number | 34 | 27 | 2 | 13 |  |  |  |  |
| TBIL  (μmol/L) | Median | 14.25 | 85.70 | - | 312.4 | ***0.0062*** | - | - | ***0.0016*** |
|  | Number | 8 | 5 | - | 8 |  |  |  |  |

EO, early-onset; LO, late-onset; AS, Asymptomatic; Lac, lactic acid；ALT, alanine aminotransferase ; AST, glutamic-oxalacetic transaminase, TBIL, total bilirubin.

Citrulline#: EO had three unspecified gender.

**S2-4** Protein-ligand binding free energy (top1) of wild-type and mutant-type structures

| No. | Mutation Site | | Free energy(kcal/mol) |
| --- | --- | --- | --- |
|  |  |  |  |
| 1 | Wild-type | | -10.94 |
| 2 | c. 959G >C | p. Arg320Pro | -9.23 |
| 3 | c.968T>G | p.Val323Gly | -9.76 |

**S2-5** PLIP results of wild-type structure

| Hydrophobic Interactions | | | | | | | |
| --- | --- | --- | --- | --- | --- | --- | --- |
| Index | Residue | AA | Distance | Ligand Atom | | Protein Atom | |
| 1 | 163A | LEU | 3.07 | 2529 | | 1008 | |
| 2 | 200A | ILE | 3.84 | 2529 | | 1305 | |
| Hydrogen Bonds | | | | | | | |
| Index | Residue | AA | DistanceH-A | DistanceD-A | Donor Angle | Donor Atom | Acceptor Atom |
| 1 | 91A | THR | 1.96 | 2.90 | 159.08 | 463 [Nam] | 2542 [O3] |
| 2 | 92A | ARG | 2.67 | 3.56 | 150321 | 470 [Nam] | 2542 [O3] |
| 3 | 92A | ARG | 1.85 | 2.74 | 148.28 | 480 [Ng+] | 2542 [O3] |
| 4 | 92A | ARG | 2.84 | 3.42 | 119.10 | 2542 [O3] | 477 [Ng+] |
| 5 | 93A | THR | 2.41 | 338 | 168.91 | 481 [Nam] | 2541 [O2] |
| 6 | 141A | ARG | 2.48 | 3.40 | 154.58 | 842 [Ng+] | 2538 [O2] |
| 7 | 141A | ARG | 1.98 | 2.90 | 153.66 | 841 [Ng+] | 2543 [O3] |
| 8 | 171A | GLN | 3.10 | 3.54 | 108.11 | 1077 [Nam] | 2538 [O2] |
| 9 | 199A | ASN | 1.58 | 2.39 | 132.66 | 2534 [N3] | 1296 [O2] |
| 10 | 199A | ASN | 1.79 | 2.73 | 160.20 | 1297 [Nam] | 2532 [O.co2] |
| 11 | 268A | MET | 2.17 | 3.13 | 165.76 | 1810 [Nam] | 2533 [O.co2] |
| 12 | 304A | LEU | 2.04 | 1.71 | 123.56 | 2536 [Nam] | 2105 [O2] |
| 13 | 330A | ARG | 2.06 | 2.80 | 130.26 | 2327 [Ng+] | 2538 [O2] |

**S2-6** PLIP results of the mutant-type(R320P) structure

| Hydrophobic Interactions | | | | | | | |
| --- | --- | --- | --- | --- | --- | --- | --- |
| Index | Residue | AA | Distance | Ligand Atom | | Protein Atom | |
| 1 | 163A | LEU | 3.6 | 2535 | | 1008 | |
| Hydrogen Bonds | | | | | | | |
| Index | Residue | AA | DistanceH-A | DistanceD-A | Donor Angle | Donor Atom | Acceptor Atom |
| 1 | 92A | ARG | 3.52 | 3.85 | 102.03 | 470 [Nam] | 2549 [O3] |
| 2 | 93A | THR | 1.99 | 2.85 | 144.76 | 481 [Nam] | 2549 [O3] |
| 3 | 93A | THR | 2.08 | 2.62 | 113.34 | 2549 [O3] | 486 [O3] |
| 4 | 141A | ARG | 1.78 | 2.74 | 163.95 | 842 [Ng+] | 2550 [O3] |
| 5 | 141A | ARG | 2.28 | 3.01 | 129.23 | 841 [Ng+] | 2545 [O2] |
| 6 | 168A | HIS | 2.62 | 3.52 | 154.46 | 2550 [O3] | 1053 [N2] |
| 7 | 199A | ASN | 1.76 | 2.73 | 169.97 | 1297 [Nam] | 2539 [O.co2] |
| 8 | 263A | ASP | 1.56 | 2.55 | 162.00 | 2541 [N3] | 1781 [O2] |
| 9 | 304A | LEU | 2.19 | 3.04 | 143.93 | 2543 [Nam] | 2112 [O2] |
| 10 | 330A | ARG | 2.72 | 3.62 | 151.89 | 2334 [Ng+] | 2549 [O3] |
| Salt Bridges | | | | | | | |
| Index | Residue | AA | Distance | Ligand Group | | Ligand Atoms | |
| 1 | 88A | LYS | 5.17 | Carboxylate | | 2539, 2540 | |

**S2-7** PLIP results of the mutant-type(V323G) structure

| Hydrophobic Interactions | | | | | | | |
| --- | --- | --- | --- | --- | --- | --- | --- |
| Index | Residue | AA | Distance | Ligand Atom | | Protein Atom | |
| 1 | 264A | THR | 3.68 | 2525 | | 1781 | |
| Hydrogen Bonds | | | | | | | |
| Index | Residue | AA | DistanceH-A | DistanceD-A | Donor Angle | Donor Atom | Acceptor Atom |
| 1 | 92A | ARG | 2.88 | 3.54 | 125.06 | 480 [Ng+] | 2538 [O3] |
| 2 | 93A | THR | 2.17 | 2.77 | 118.13 | 481 [Nam] | 2537 [O2] |
| 3 | 93A | THR | 2.82 | 3.50 | 127.42 | 2539 [O3] | 486 [O3] |
| 4 | 141A | ARG | 1.81 | 2.56 | 130.33 | 841 [Ng+] | 2539 [O3] |
| 5 | 141A | ARG | 2.67 | 3.39 | 130.24 | 842 [Ng+] | 2534 [O2] |
| 6 | 198A | ASN | 3.48 | 3.85 | 104.86 | 1289 [Nam] | 2530 [N3] |
| 7 | 199A | ASN | 2.35 | 3.26 | 153.02 | 1297 [Nam] | 2528[O.co2] |
| 8 | 263A | ASP | 2.23 | 3.08 | 138.88 | 2530 [N3] | 1773 [O3] |
| 9 | 268A | MET | 1.95 | 2.90 | 162.62 | 1810 [Nam] | 2529 [O.co2] |
| 10 | 303A | CYS | 1.98 | 3.85 | 149.90 | 2538 [O3] | 2099 [O2] |
| 11 | 304A | LEU | 2.13 | 2.83 | 126.62 | 2532 [Nam] | 2105 [O2] |
| 12 | 330A | ARG | 1.81 | 2.54 | 128.37 | 2323 [Ng+] | 2537 [O2] |
| 13 | 330A | ARG | 3.41 | 3.86 | 110.01 | 2324 [Ng+] | 2537 [O2] |
